# Muscimol injection into the ventral posterolateral nucleus of the thalamus impairs tactile reward-seeking behavior but preserves affective vocalization in male rats

**DOI:** 10.1371/journal.pone.0351495

**Published:** 2026-06-10

**Authors:** Rie Shimoju

**Affiliations:** Center for Basic Medical Research, International University of Health and Welfare, Tochigi, Japan; University of Illinois at Urbana-Champaign, UNITED STATES OF AMERICA

## Abstract

Ultrasonic vocalizations (USVs) are a useful tool for evaluating emotion and motivation in rodents. Rhythmic stroking in rats induces positive affective 50-kHz USVs and reward-seeking behavior. This response involves the dorsal column tract as the ascending pathway; however, supraspinal mechanisms remain unknown. We hypothesized that the ventral posterolateral nucleus of the thalamus (VPL), which receives somatosensory inputs from the spinal cord via the dorsal column–medial lemniscus pathway, might be involved in positive 50-kHz USV production and motivated tactile reward-induced behavior. To test the hypothesis, we used young adult male rats before and after inactivation of the VPL with bilateral infusion of muscimol, a potent γ-aminobutyric acid (GABA)-A type receptor agonist. We measured 50-kHz USVs, approach latency, and spontaneous behaviors pre- and post-muscimol injection, focusing on two emotional (during rhythmic stroking and receiving reward) and motivational (after rhythmic stroking and reward-seeking) conditions. Increased GABAergic inhibition in the VPL completely impaired approach behavior without causing obvious sensory and motor dysfunctions, but only slightly affected 50-kHz USVs. The present data indicate that the GABAergic system in the VPL is substantially involved in execution of reward-seeking behavior but not in the 50-kHz USVs induced by tactile rewards.

## Introduction

Rats are highly vocal animals, primarily using ultrasonic vocalizations (USVs), which are widely validated, noninvasive behavioral biomarkers used in research to assess emotion, motivation, and underlying neural mechanisms [1–13]. Specifically, juvenile and adult rats emit two primary types of USVs, proposed to indicate positive and negative emotional states. The 22-kHz USVs are observed in negative emotional states, such as predator exposure, social defeat, restraint, and gentle touch by an unfamiliar human hand, and foot shock [14–20]. These calls are accompanied by fear- and anxiety-related behaviors such as immobilization and freezing [18,21,22].Conversely, 50-kHz USVs have been reported during copulation, food anticipation, psychostimulant administration, play behavior, tickling, gentle back stroking, and rhythmic stroking [23–29]. These calls are associated with positive behaviors such as approach and locomotion [11,26,30,31].

Although it has been proposed that rat 50-kHz USVs are a reliable indicator of positive emotions, important issues have been pointed out when analyzing and interpreting USVs [10]. One important issue is that the classification of rat USVs is diverse, new types of USVs are currently being reported, and new classification methods for 50-kHz USVs are still being reported [32–37], resulting in a lack of a definitive classification method [10]. However, the wide variety of classifications of 50-kHz USVs is understandable for the following reasons: 50-kHz USV subtypes are significantly influenced by strain, sex, age, and stimulus [28,29,38–41], and experimental conditions, making it difficult to adopt a uniform classification across all laboratories. Therefore, while classification methods seem to be currently left to the discretion of individual researchers, efforts are needed to develop simpler and more widely accepted classification methods that would facilitate shared interpretation of results and lead to a deeper understanding of 50-kHz USVs. In any case, the simplest and traditional classification is to divide 50-kHz USVs into frequency-modulated (FM) and flat calls based on their frequency and sonogram features [3,42], and the detailed classification method is the classification by Wright et al. [43], which is a benchmark for subsequent studies [10]. Wright et al.’s classification involves visually classifying spectrogram features, so obtaining sonograms with high visibility is important to increase the reliability of the results. Detailed classification of 50-kHz USVs has the advantage of increasing the amount of information available but it also increases the complexity of data analysis and may place an excessive burden on researchers [10]. Therefore, our laboratory developed a somatosensory stimulation method that minimizes noise and allows clear sonograms to be obtained by elevating the rat and maintaining relatively constant microphone and head positions during stimulation [28]. We have sought to simplify the classification of Wright et al. [43] by examining the characteristic vocalizations observed during and after stimulation, based on factors such as their occurrence frequency, correlation with behavior and EEG [11,28,31], and the differences in the effects of dopamine receptor blocker administration in the nucleus accumbens [13] and dorsal column lesions [12]. Therefore, in this study, we used a simpler classification method based on the classification by Wright et al. [43], which has been improved and adopted in our laboratory [11–13,28,31]. We also used the simplest classification method, which divides USVs into two types: FM and flat, to help determine whether simplification of classification is possible.

The somatosensory system is an essential physiological function for animal survival, as it enables smooth, coordinated movement through the perception of the external world and proprioception. Further, the somatosensory system plays a crucial role in higher brain functions such as emotion processing [44–47]. The ascending somatosensory pathways that originate from the spinal cord transmit various modalities of somatosensory information to several brain areas that are involved in emotion, motivation, and reward processing [48].

Human and animal research has revealed that tactile stimulation, which is one of the somatic sensations, is associated with positive emotions. Numerous human studies confirm that touch, particularly gentle stroking, significantly reduces stress and anxiety and induces pleasant emotion [49–51]. We previously reported that tactile stroking induced 50-kHz USVs that reflect positive affective states in rats [28]. In our research, we developed a rat model of positive emotion and motivation induced by somatosensory stimulation in young adult rats. That is, during rhythmic stroking, the rats produce abundant 50-kHz USVs that are mediated by the mesolimbic accumbal dopamine system [13]. In our previous study, we revealed that, after rhythmic stroking, the exploratory behaviors of rats increased when the experimenter’s hand was not present in the cage, whereas they quickly approached the hand, clearly demonstrating goal-directed tactile reward-seeking behavior when the experimenter’s hand was present in the cage [12,31]. Thus, rhythmic stroking can be interpreted as having reward (i.e., emotional output characterized by 50-kHz USVs during stimulation) and motivational values (i.e., motivational output characterized by motivational actions accompanied with 50-kHz USVs after stimulation). As mentioned above, the mesolimbic accumbal dopamine system, involved in rhythmic stroking induced 50-kHz USVs [13], plays a crucial role in processing reward and motivation and regulating reward-directed approach [52,53]. Therefore, somatosensory-emotion and/or motivation systems may be activated during and after rhythmic stroking. However, the brain structures that relay sensory information to the positive emotional and/or motivational systems remain largely unidentified.

Tactile stimulation, such as stroking, was the most effective way in our study to induce 50-kHz USVs compared with swinging, which is considered a vestibular and proprioceptive stimulus, and light touch without stroking [28]. Accordingly, it is hypothesized that somatosensory information from the mechanoreceptors activated by tactile movement ascends the spinal cord and projects to the brain and is involved in modality segregation, emotion, and motivational behavior. Somatosensory information from the body is transmitted from the periphery to the cerebral cortex through different ascending pathways [48]. We have recently reported the involvement of somatosensory signals, transmitted by the dorsal column tract, in inducing affective 50-kHz USVs and approach (goal-directed reward-seeking behavior) [12]. The ascending fibers from the dorsal column nuclei (DCN) terminate in the ventral posterolateral nucleus of the thalamus (VPL) [54]. A primate study reported that VPL is involved not only in sensory processing (e.g., modality segregation) [55] but also in decision-making and goal-directed behavior [56–58]. However, knowledge about the role of the VPL in reward and motivation in rodents is limited.

Siviy and Panksepp [59] revealed that electrolytic lesion of the ventrobasal complex, comprising the VPL and the ventral posteromedial nucleus of the thalamus (VPM), exhibited minimal effects on rough-and-tumble play, which required somatosensory inputs and motivation to play in juvenile rats. In their study, 50-kHz USVs were not assessed. Further, electrolytic lesions employed in this study can destroy nerve fibers passing through [60]; therefore, the effects of the lesion may be more widespread than the ventrobasal complex. There is a clear difference in the mechanisms that trigger motivation between rhythmic stroking, which is extrinsically rewarding, and intrinsically motivated rough-and-tumble play [61]. Further, various sensory information, including proprioceptive, vestibular, and visual changes, may be involved in inducing changes in emotion and motivation considering the drastic postural changes during rough-and-tumble play. Hence, the involvement of the VPL in the expression of positive 50-kHz USVs and reward-seeking behavior caused by extrinsic somatosensory stimulation remains unclear.

VPL thalamocortical neurons are mainly glutamatergic [54,62], and the VPL has no GABAergic interneurons in rats [63–65]; whereas, VPL neurons do possess GABA receptors [64,66], which are activated by GABAergic input mainly from the reticular nucleus of the thalamus (RT) [62]. This acts as a critical emotional gating between the thalamus and the cortex to modulate sensory processing and attention [67–72]. The RT provides GABAergic modulation to the VPL and has altered the strength of inhibitory input to VPL neurons [73]. Specifically, GABA_A_ receptor-mediated inhibitory postsynaptic currents have been recorded in VPL neurons [74]. Further, enhanced GABAergic synaptic input to the VPL has contributed to somatosensory thalamic dysfunction [75]. Therefore, GABAergic projection from the RT may be crucial for the proper VPL functioning in the thalamocortical system, which is involved in higher brain functions, including emotion and motivation. Thus, we hypothesize the involvement of the VPL GABAergic system in regulating tactile reward-induced affective vocalizations and motivated behaviors. To test this hypothesis, we have investigated the effect of GABA_A_ receptor agonist muscimol injections into the bilateral VPL on affective vocalizations and motivated behaviors in rats. To date, no studies have clarified whether the VPL plays a role in USV generation as part of a somatosensory afferent pathway. The importance of this study lies in its contribution to identifying the neural circuits that underlie positive emotions and motivational behaviors elicited by tactile stimulation.

## Materials and methods

All animal experiments were approved by the Ethics Committee of Animal Experiments of the International University of Health and Welfare (Otawara, Japan; Permission Number: 25004). All experimental procedures and protocols were conducted with the Japanese Physiological Society’s Guide for the care and use of animals in the field of physiological sciences. All efforts were made to minimize animal suffering and distress. A skilled experimenter performed all surgeries and animal handling.

### Animals

The sample size calculation was performed in G*Power (version 3.1.9.7) based on an alpha error of 0.05 and 80% power, with at least eight rats per group required for repeated analysis of variance (ANOVA) measures. This study used 12 male Wistar/ST rats (7 weeks old, 210–230 g at the time of surgery) (Japan SLC, Inc., Shizuoka, Japan). Only male rats were included to exclude the influence of sex differences and estrous cycles, which are known to affect USVs and reward-related behaviors [41,76,77]. This decision was also based on our previous experiments that focused on male rats to investigate the mechanisms underlying USV production during rhythmic stroking stimulation, a paradigm established in our laboratory [11–13,28,31]. The experiment was conducted in three sessions. In the first session, four rats were used: two for preliminary experiments to establish the surgical procedure and drug concentration (their USVs were excluded from behavioral analysis), and two for data collection. In the second and third sessions, four rats were used in each, yielding eight additional rats for data collection. Thus, a total 10 rats contributed to the dataset (n = 10). The experimental environment and protocol were consistent across sessions, with stimulation, data recording, and analysis performed by the same experimenter. Because the study employed a within-subject design, all rats underwent both vehicle and muscimol administration experiments. Each experiment lasted three weeks, resulting in a total study duration of 12 weeks. Post-arrival, animals were housed in pairs in standard polycarbonate cages (width 27 cm × length 44 cm × height 18 cm). Animals were maintained under controlled temperature (23℃ ± 1℃) and light cycle (12-h/12-h light/dark, light on at 08:00 h). Standard rodent food (Labo-MR stock, Nosan Corporation, Kanagawa, Japan) and water were provided ad libitum. After 1 week of acclimation to the laboratory environment, rats were individually housed and handled for 2 min daily for 1 week preoperatively, based on our previous study [31]. USVs, behavioral activities, and approach latency were measured in rats preoperatively and 7 days postoperatively pre- and post-muscimol (see paragraph Microinjection of the GABA_A_ receptor agonist below) or vehicle microinjection to the bilateral VPL. All experiments were conducted between 09:00 and 16:00.

### Surgery

Animals were anesthetized with sodium pentobarbital (40 mg/kg, intraperitoneally) and maintained with isoflurane. Rats were mounted on a stereotaxic apparatus in the flat skull position. Guide cannulas (0.35 mm inner diameter [i.d.], 0.5 mm outer diameter [o.d.]; Eicom, Kyoto, Japan) were implanted bilaterally into the VPL (3.0 mm posterior, ± 3.5 mm lateral to bregma and 3.0 mm below the dura), which is an area that presents large cutaneous receptive fields on the trunk [78] following the stereotaxic atlas of Paxinos and Watson [79]. The skull was secured with stainless steel screws and dental cement. Dummy cannulas (0.35 mm o.d., Eicom, Kyoto, Japan) were inserted into guide cannulas to prevent possible clogging. A preventive antibiotic and analgesic therapy was administered preoperatively (Carprofen 50 mg/kg, subcutaneously; Zoetic Japan Inc., Tokyo, Japan). Postoperatively, rats received a subcutaneous injection of 1% lidocaine (Sandoz AG, Tokyo, Japan) and an antibiotic (penicillin G procaine, 40,000 units/kg, intramuscularly; Riken Vets Pharma Inc., Saitama, Japan). All rats were allowed to recover from anesthesia on a heating pad before returning to their cages. Their health and behavior were monitored daily until the day of the experiment.

### Microinjection of GABA_A_ receptor agonist

On the day of the experiment, injection cannulas (0.35 mm o.d., Eicom, Kyoto, Japan) were inserted into the bilateral VPL through the guide cannula. The injection cannula, which is 2 mm lower than the guide cannula, was used for microinjection with a polyethylene catheter connecting a gastight syringe. GABA_A_ receptor agonist muscimol (50 ng/0.2 µL per side, Sigma-Aldrich, St. Louis, MO, USA) or vehicle (physiological saline 0.2 µL) was injected into the VPL on both sides over 2 min using a microsyringe pump (ESP-32, Eicom, Kyoto, Japan). The injection cannula was held on for at least 2 min to permit drug diffusion. Behavior tests were conducted 30 min after drug/vehicle injection. The muscimol dose used in this study is commonly utilized to inactivate limbic and thalamic nuclei [80–83].

### Rhythmic stroking

Rhythmic stroking was conducted based on our previous study [12,13,28]. In brief, each rat was gently held by the back skin and repeatedly (approximately 1–1.5 Hz) stroked by the experimenter’s bare hand on its ventral side for 30 s at a speed of 15–20 cm/s.

### Recording and analysis of USVs

We recorded and analyzed USVs as previously described [12,13,31]. In brief, an UltraSoundGate 116H audio device with a CM16/CMA microphone (Avisoft Bioacoustics, Berlin, Germany) was used to record USVs. The recordings were transferred to SASLab Pro (version 5.3.2, Avisoft Bioacoustics) for acoustic analyses. Further, 50-kHz USVs were analyzed manually following Wright *et al*.’s classification [43] and our previous study using rhythmic stroking [11–13]. In the present study, 50-kHz USVs were divided into four categories: flat, harmonics, trill + complex + step up, and other FM calls (S1 Fig). In our previous research, we defined harmonics (predominantly flat calls with harmonics) as a subtype of overtones other than split and composite [11–13,31]. The split was classified according to Wright et al.’s classification [43], and consists of three parts; only the middle part contains harmonics. In this study, split calls and predominantly flat calls with harmonics (harmonic flat, step down with harmonics, and step up with harmonics) [13] were classified as the same type of calls and expressed as harmonics since all have harmonic overtones, are present during rhythmic stroking, disappear after stimulation [11–13,28,31], and are reduced by dorsal column lesions [12]. In addition, we used a widely accepted two-category classification: FM and flat calls (with/without overtones) [42].

### Approach latency

We measured the approach latency based on previous studies [31,84–86]. After recording USVs for the first 30 s of stimulation and post-stimulation (30 s), the rats received a second 30-s stimulation. Afterward, they were immediately placed in the corner of the home cage. Approach latency was measured as the time from this moment until the rats approached and touched the experimenter’s hand. The maximum latency was set to 30 s. In this study, approaching the experimenter’s hand is considered a goal-directed reward-seeking behavior.

### Behavioral recording and analysis

Pre- and post-drug injection, spontaneous behaviors were observed to detect any motor deficit caused by muscimol/vehicle injection. Behavioral recording and analysis were conducted as previously described [11,12]. In brief, behaviors were monitored using a digital video camera (HDR-CX680, SONY, Tokyo, Japan) while recording USVs. After the experiment, behavioral activities were manually scored offline by counting the number of occurrences and measuring the target behavior duration. The following behavior categories were distinguished during observation: locomotion (walking and running), rearing (either free-standing or against a wall), and exploring (standing against the wall and sniffing). The total count (for rearing) or total duration (locomotion and exploring) was measured as behavioral parameters after rhythmic stroking.

### Experimental protocols

Tactile reward-induced 50-kHz USVs are stable across days in the same animal but highly variable among individuals [31,87]; thus, we used a within-subject design in our study. After a 1-week handling period, the rats underwent surgery to implant guide cannulas. Acoustic and behavioral parameters for each animal were performed preoperatively. Seven days postoperatively, acoustic and behavioral parameters were made before and after muscimol/vehicle injection. Each animal received one dose of either vehicle or muscimol once a day. Rhythmic stroking was applied for 30 s at 5 min pre-muscimol/vehicle injection (control condition) and 30 min post-injection. In all the experiments, we simultaneously recorded USVs and behavioral activity continuously for 90 s (30 s baseline, 30 s stimulation, and 30 s poststimulus). Immediately after 90 s of USV data recording, 30-s stimulation was re-applied to measure the approach behavior observed post-stimulation. USV data were assessed before (30 s, baseline), during (30 s), and after (30 s) rhythmic stroking.

### Histology

After completing the experiments, rats were deeply anesthetized with sodium pentobarbital (i.p., 100 mg/kg) and received injections of 2% Evans Blue (EB) solution into bilateral VPL using the same injection volume and speed utilized in the experiment. All rats were then intracardially perfused with heparinized saline, followed by 4% formaldehyde in 0.1 M phosphate-buffered saline. The brain was removed and cryoprotected in a 30% sucrose solution. The brain was sliced into 50-μm-thick serial sections using a cryostat (Leica CM3050 S; Leica Biosystems, Nussloch, Germany). Sections were stained with cresyl violet and assessed microscopically to verify the location of the cannula injection site in the VPL by referring to the atlas of Paxinos and Watson [79].

### Statistical analysis

Data are expressed as mean ± standard error of the mean. The Shapiro–Wilk test was conducted to check the normality of the datasets. Because measurements were obtained repeatedly from the same animals, all relevant analyses were conducted using repeated-measures designs. Normally distributed data were subjected to parametric tests (three-way ANOVA followed by post hoc Bonferroni tests for the effects of the drug [muscimol or vehicle] × treatment [pre- or post-injection] × time course [before, during, and after stimulation] measurements; two-way ANOVA followed by post hoc Bonferroni tests for the effects of the treatment [pre- or post-injection] × time course [before, during, and after stimulation] measurements; one-way ANOVA followed by post hoc Bonferroni tests for time course measurements and paired *t*-test for group comparisons). Non-normally distributed data were subjected to nonparametric tests (Friedman test followed by a post hoc Dunn–Bonferroni test for repeated measures and the Wilcoxon signed-rank test for group comparisons). Statistical Package for the Social Sciences version 23 (IBM Cor, Armonk, NY, USA) was used for all statistical analyses. In all analyses, *p*-values of <0.05 (two-tailed) indicated statistical significance.

## Results

### Histological verification of injection sites

**Fig 1:**
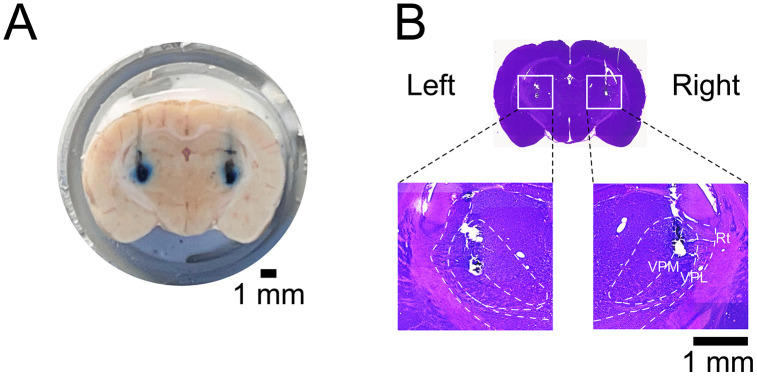
Histological verification of all injection sites of bilateral VPL. (A) Photograph showing the diffusion area of Evans Blue (EB) at the injection sites. (B) Photomicrograph of a coronal section of the rat brain illustrating cannula placements of bilateral injections into the VPL in one representative animal.

We mainly focused on the dorsal portion of the middle VPL, as it receives mainly cutaneous input from the trunk [78]. We used EB dye injected at the same volume (0.2 μL) as the muscimol or vehicle solutions to identify its diffusion (Fig 1). The assessment of the extent of EB diffusion in all rats based on experimental data confirmed that EB dye mainly spread to the target area of the VPL.

### Bilateral muscimol injection into the VPL preserves affective 50-kHz USVs during and after rhythmic stroking

**Fig 2:**
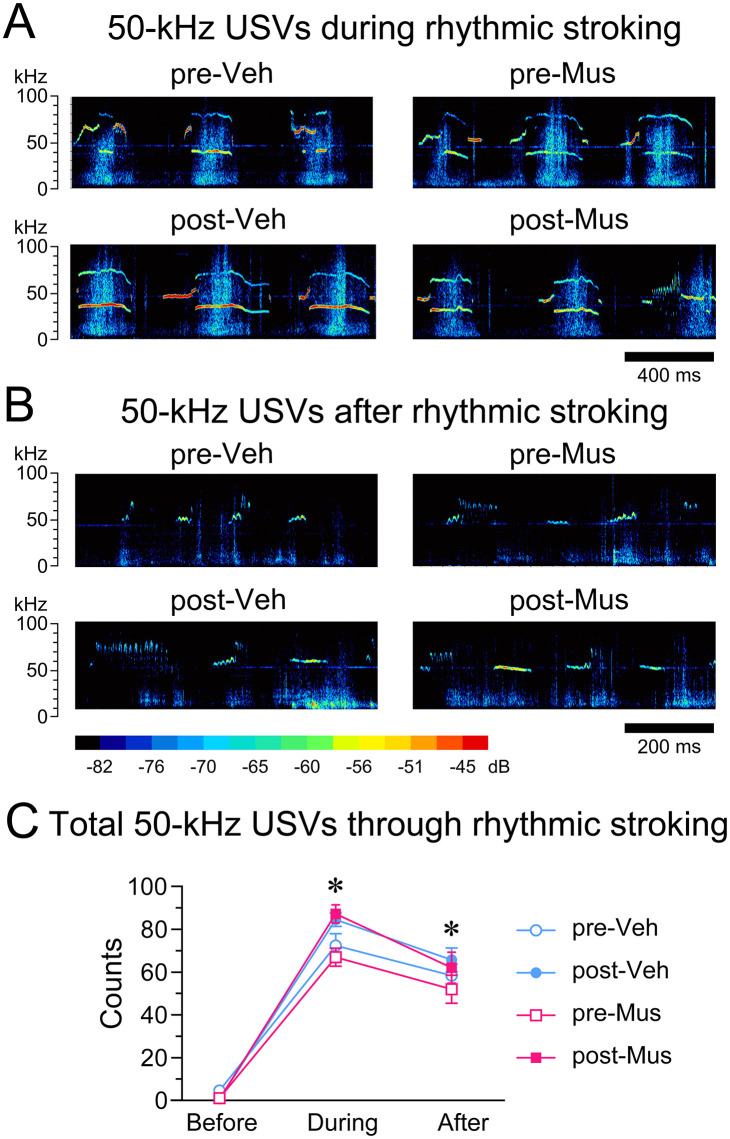
Muscimol injection into the VPL did not alter the total number of 50-kHz USVs. Representative spectrograms of 50-kHz USVs during (A) and after (B) rhythmic stroking in a representative rat pre- and post-vehicle/muscimol injection. (C) Total number of 50-kHz USVs before, during, and after rhythmic stroking pre- and post-vehicle (Veh) or muscimol (Mus) injection. * *p* < 0.0001 compared to before stimulation using one-way RM-ANOVA followed by post hoc Bonferroni tests for pairwise comparisons. N = 10.

To investigate whether activation of GABAergic system in the VPL could affect emotion and motivation induced by somatosensory stimulation, we recorded 50-kHz USVs and its subtypes during and after rhythmic stroking. Fig 2A and 2B show representative sonograms of 50-kHz USVs pre- (control) and post-injection in vehicle or muscimol groups during and after rhythmic stroking. Bilateral injection of a GABA_A_ receptor agonist muscimol into the VPL did not change the number of 50-kHz USVs both during and after stroking compared with vehicle treatment (Fig 2C). Three-way ANOVA analysis revealed no significant main effect of the drug (i.e., muscimol or vehicle) on the total number of 50-kHz USVs (F[1, 36] = 1.04, *p* > 0.05, *η*^2^_*p*_ = 0.104) but revealed a significant main effect of treatment (i.e., pre- or post-injection) (F[1, 36] = 12.146, *p* = 0.007, *η*^2^_*p*_ = 0.574] and time (i.e., before, during, or after treatment) (F[2, 36] = 195.862, *p* = 0.0001, *η*^2^_*p*_ = 0.956) on 50-kHz USVs production. There was no significant no significant drug × treatment interaction (F[1, 36] = 1.056, *p* > 0.05, *η*^2^_*p*_ = 0.105), drug × time interaction (F[2, 36] = 0.270, *p* > 0.05, *η*^2^_*p*_ = 0.029), or drug × treatment × time interaction (F[2, 36] = 0.175, *p* > 0.05, *η*^2^_*p*_ = 0.019), but there was a significant treatment × time interaction (F[2, 36] = 6.327, *p* = 0.008, *η*^2^_*p*_ = 0.413). Likewise, we observed a significant main effect of time on the total number of 50-kHz USVs in both vehicle-injected and muscimol-injected rats (two-way ANOVA; Veh: F[2, 18] = 131.5, *p* < 0.0001, *η*^2^_*p*_ = 0.936; Mus: F[2, 18] = 139.318, *p* < 0.0001, *η*^2^_*p*_ = 0.939). One-way ANOVA revealed a significant main effect of time on the total number of 50-kHz USVs with rhythmic stroking in both vehicle-injected and muscimol-injected rats [pre-Veh: F(2, 9) = 71.844, *p* < 0.0001, *η*^2^_*p*_ = 0.889; post-Veh: F(2, 9) = 154.283, *p* < 0.0001, *η*^2^_*p*_ = 0.945; pre-Mus: F(2, 9) = 78.217, *p* < 0.0001, *η*^2^_*p*_ = 0.897; post-Mus: F(2, 9) = 74.041, *p* < 0.0001, *η*^2^_*p*_ = 0.892]. Rhythmic stroking significantly increased the number of total 50-kHz USVs during (pre-Veh: 75.1 ± 5.8 calls, *p* < 0.0001; post-Veh: 87.7 ± 3.3 calls, *p* < 0.0001; pre-Mus: 69.4 ± 4.4 calls, *p* < 0.0001; post-Veh: 90.5 ± 4.4 calls, *p* < 0.0001) and after (pre-Veh: 60.5 ± 5.6 calls, *p* < 0.0001; post-Veh: 68.2 ± 5.8 calls, *p* < 0.0001; pre-Mus: 53.9 ± 6.8 calls, *p* < 0.0001; post-Veh: 64.4 ± 7.5 calls, *p* < 0.0001) stimulation compared to baseline (pre-Veh: 4.8 ± 2.1 calls; post-Veh: 0.7 ± 0.3 calls; pre-Mus: 1.0 ± 0.8 calls; post-Veh: 0.7 ± 0.4 calls; Fig 2C).

Furthermore, no significant differences were observed in the total number of 50-kHz USVs during or after rhythmic stroking before and after guide cannula implantation surgery [one-way ANOVA: during rhythmic stroking, F(2, 27) = 0.326, *p* > 0.05, *η*^2^_*p*_ = 0.035; after rhythmic stroking, F(2, 27) = 1.415, *p* > 0.05, *η*^2^_*p*_ = 0.136] (S2 Fig).

### Bilateral muscimol injection into the VPL only slightly affected the categorized 50-kHz USVs

Acoustic analyses were conducted on 6,255 calls from ten rats during and after stimulation (during stimulation: 751, 876, 694, and 904 calls in pre-Veh, post-Veh, pre-Mus, and post-Mus, respectively; after stimulation: 605, 682, 540, 644, and 559 calls in pre-Veh, post-Veh, pre-Mus, and post-Mus without hand, and post-Mus with hand, respectively). S1 Fig shows representative sonograms of categorized 50-kHz USVs subtypes in vehicle groups during and after rhythmic stroking. In the traditional simple classification into FM and flat types (S3 Fig), we observed no significant main effect of drug and treatment on the number of flat calls (two-way ANOVA; drug: F[1, 18] = 2.412, *p* > 0.05, *η*^2^_*p*_ = 0.211; treatment: F[1, 18] = 4.395, *p* > 0.05, *η*^2^_*p*_ = 0.328) and no significant drug × treatment interaction (F[1, 18] = 0.376, *p* > 0.05, *η*^2^_*p*_ = 0.04). However, we observed a significant main effect of treatment (two-way ANOVA; F[1, 18] = 10.724, *p* = 0.010, *η*^2^_*p*_ = 0.544) but not of drug (two-way ANOVA; F[1, 18] = 0.211, *p* > 0.05, *η*^2^_*p*_ = 0.023) on the number of FM calls and no significant drug × treatment interaction (F[1, 18] = 1.211, *p* > 0.05, *η*^2^_*p*_ = 0.119). Flat calls after rhythmic stroking significantly increased in post-Mus compared with pre-veh (χ^2^ = 1.962, *p* = 0.050) and pre-Mus (χ^2^ = 2.524, *p* = 0.012). Flat calls did not change in post-veh compared with pre-veh (χ^2^ = 1.063, *p* > 0.05). We did not observed a significant main effect of drug (two-way ANOVA; F[1, 18] = 3.029, *p* > 0.05, *η*^2^_*p*_ = 0.252) and treatment on FM calls after rhythmic stroking (two-way ANOVA; F[1, 18] = 0.496, *p* > 0.05, *η*^2^_*p*_ = 0.052) and no significant drug × treatment interaction (F[1, 18] = 0.527, *p* > 0.05, *η*^2^_*p*_ = 0.046).

**Fig 3:**
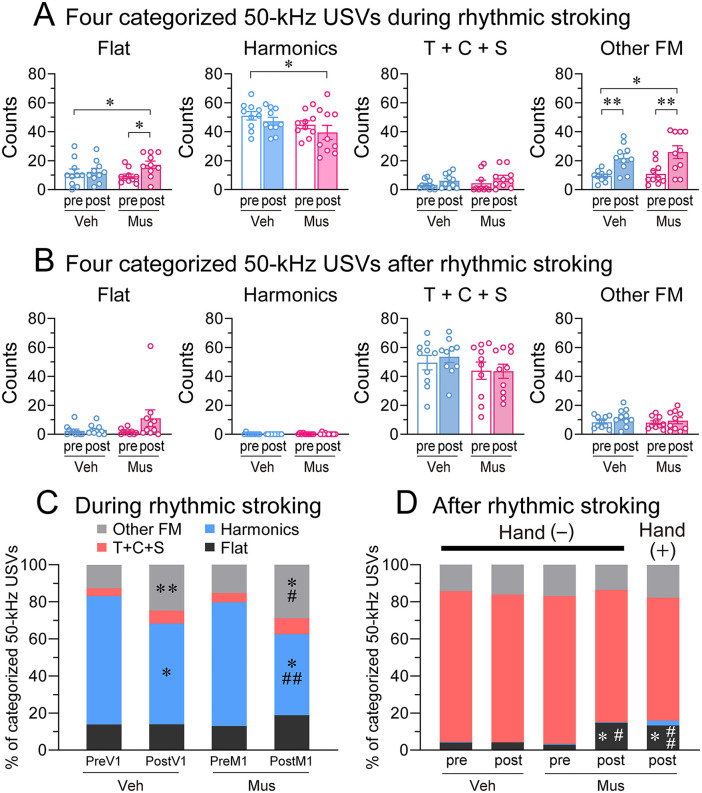
Muscimol injection into the VPL slightly alters categorized 50-kHz USVs during and after rhythmic stroking. The number of 4-category classification of 50-kHz USVs during (A) and after (B) rhythmic stroking in rats pre- and post-injection. The percentage occurrence rate of categorized 50-kHz USVs during (C) and after (D) rhythmic stroking pre- and post-vehicle (Veh) or muscimol (Mus) injection. In (D), USVs were recorded both without and with the experimenter’s hand in the cage, as represented by hand (−) and hand (+), respectively. * *p* < 0.05, ** *p* < 0.01 compared with pre-vehicle injection using the paired t-test or Wilcoxon signed-rank test. ^#^ *p* < 0.05, ^##^ *p* < 0.05 compared with pre-muscimol injection using the paired t-test or Wilcoxon signed-rank test. N = 10.

We also observed subtle differences in 4-category classification of 50-kHz USVs between vehicle- and muscimol-injected animals during rhythmic stroking (Fig 3A). Specifically, we observed a significant main effect of treatment on the number of flat and other FM calls (two-way ANOVA; flat calls: F[1, 18] = 6.337, *p* = 0.033, *η*^2^_*p*_ = 0.413; other FM calls: F[1, 18] = 19.801, *p* = 0.002, *η*^2^_*p*_ = 0.688). The flat calls were increased in post-Mus compared with pre-Veh and post-Mus during rhythmic stroking (*t* = −2.429, *p* = 0.038, *t* = −2.308, *p* = 0.046, respectively). The numbers of other FM calls were significantly increased post-drug injection in both vehicle (*t* = −3.865, *p* = 0.004) and muscimol (*t* = −3.714, *p* = 0.005) injection. We conducted the same analysis after rhythmic stroking and revealed no significant modification between pre- and post-injection of either vehicle or muscimol (Fig 3B).

To further analyze the effects of muscimol treatment on acoustic variations provoked by rhythmic stroking, we expressed them as the percentage of occurrence of each subtype relative to the total number of occurrence for each rat (Fig 3C). We observed a significant main effect of treatment on the proportion of harmonics and other FM calls during rhythmic stroking (Friedman test; harmonics calls: χ^2^ = 15.240, *p* = 0.002, two-way ANOVA; other FM calls: F[1, 18] = 12.884, *p* = 0.006, *η*^2^_*p*_ = 0.589). Significantly higher frequencies of other FM calls were observed during rhythmic stroking in both post-Veh compared with pre-Veh (*t* = −3.392, *p* = 0.008) and post-Mus compared with pre-Mus (*t* = −2.702, *p* = 0.024). Harmonics calls were significantly decreased in post-Veh compared with pre-Veh during rhythmic stroking (*t* = −2.395, *p* = 0.017). Harmonics calls were significantly reduced in post- Veh compared with pre-Veh during rhythmic stroking (*t* = −2.395, *p* = 0.017) and post-Mus compared with pre-Veh (*t* = −2.497, *p* = 0.013). Finally, we also compared the proportion of each subtype after stroking during approach latency (Fig 3D). After rhythmic stroking, flat calls increased in both post-Mus without hand and with hand compared with pre-Veh (χ^2^ = 2.547, *p* = 0.011, χ^2^ = 2.090, *p* = 0.037, respectively) and compared with pre-Mus (χ^2^ = 2.429, *p* = 0.015, χ^2^ = 2.701, *p* = 0.007, respectively).

### Bilateral muscimol injection into the VPL totally inhibited approach behavior after rhythmic stroking without distinct sensory and motor deficient

**Fig 4:**
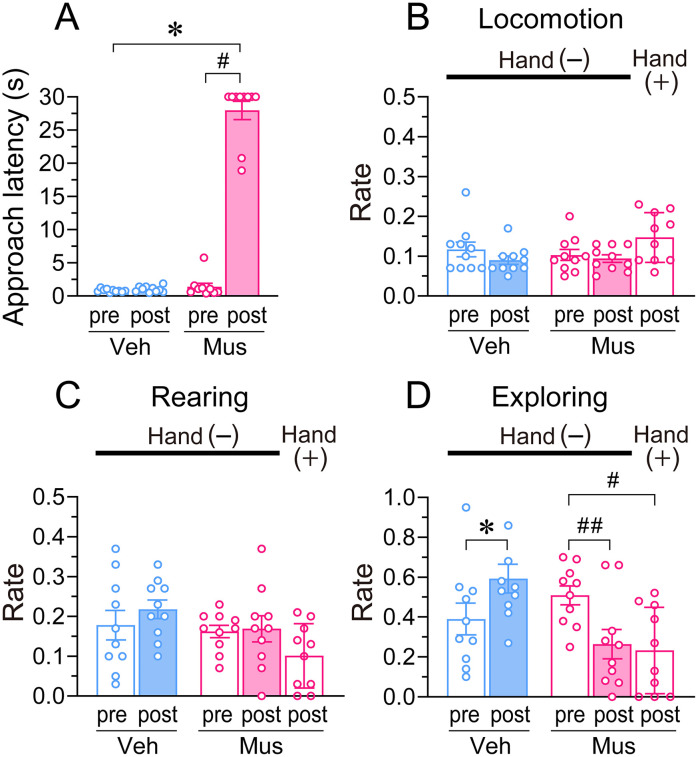
Muscimol injection into the VPL markedly suppressed approach behavior without affecting locomotion. (A) Approach latency after rhythmic stroking in rats pre-and post-injection. (B) Locomotion duration per second. (C) Rearing counts per second. (D) Exploring duration per second. In (B, C, and D), behaviors were recorded both without and with the experimenter’s hand in the cage as represented by hand (−) and hand (+), respectively. ^*^ *p* < 0.05 compared with pre-Veh, ^#^ *p* < 0.05, ^##^ *p* < 0.01, compared with pre-Mus; significant difference based on the Wilcoxon signed-rank test. N = 10.

To determine whether inactivation of the VPL could affect goal-directed motivated behavior induced by somatosensory stimulation, we measured approach latency after rhythmic stroking. We also investigated spontaneous behaviors to investigate if muscimol had any effect on motor function. The approach latency was short both pre- (0.8 ± 0.1 s) and post-vehicle (1.0 ± 0.1 s) administration, with no significant difference between the two groups (*t* = 1.800, *p* > 0.05) (Fig 4A). By contrast, muscimol-treated rats demonstrated a significant increase in approach latency (post-Mus: 28.0 ± 1.4 s) compared with pre-Veh (χ^2^ = −4.070, *p* < 0.0001) and pre-Mus (1.4 ± 0.5 s, χ^2^ = −3.204, *p* = 0.008). In the post-Mus condition, two rats exhibited minor functional deficits in the forepaw, with mild paw-slips during rearing against the wall; however, there was no ataxia or postural control dysfunction. All rats were able to walk, and no distinct change was observed in locomotion and rearing induced by rhythmic stroking pre- and post-muscimol injection into the VPL, although exploratory behavior was significantly affected by muscimol injection (Fig 4B, C; locomotion: χ^2^ = 8.529, *p* > 0.05; rearing: χ^2^ = 8.649, *p* > 0.05; exploring: χ^2^ = 23.030, *p* < 0.0001). Exploratory behavior was significantly increased in post-Veh compared with pre-Veh (Fig 4D; *z* = 2.547, *p* = 0.011). Exploratory behavior was significantly decreased in post-Mus with and without the experimenter’s hand compared with pre-Mus (Fig 4D; post-Mus without hand: *z* = −2.599, *p* = 0.009; post-Mus with hand: *z* = −2.497, *p* = 0.013), whereas it was significantly increased in post-Veh compared with pre-Veh (Fig 4D; *z* = 2.547, *p* = 0.011).

## Discussion

In this study, we investigated the involvement of the VPL in positive 50-kHz USVs and reward-seeking motivated behaviors induced by rhythmic stroking. GABA_A_ receptor agonist injected into the bilateral VPL totally impaired the reward-seeking behavior but had a subtle effect on positive 50-kHz USVs. Present results indicate that the GABAergic system in the VPL plays a crucial role in motivated behavior induced by somatosensory signals, but is not necessarily involved in inducing positive emotion in response to rhythmic stroking. These data indicate a dissociation of somatosensory processing between the sensory-emotion and sensory-motivation systems and the involvement of VPL in the mechanisms. Siviy and Panksepp [88] showed that lesions of the dorsomedial thalamic nucleus or the parafascicular nucleus of the thalamus differentially affected play behavior and the motivation to play in juvenile rats, suggesting that the thalamus may differentially regulate positive affect and motivation. Furthermore, it has been reported that thalamic dysfunction clearly affects attentional processes while sparing emotion processing [89]. These findings supported our results, demonstrating that thalamo-somatosensory dysfunction using muscimol impaired motivation but spared emotion system. Our results may support the idea that the thalamus functions as a bridge between perception, cognition, and emotion [90]. Moreover, thalamo-somatosensory/parietal connectivity is considered a potential marker of general psychiatric disorder [91].It has been systematically reviewed that analysis of 50-kHz USVs has become a widely used tool in rat behavioral science, both in basic research and in the study of neuropsychiatric disorders [10]. The results of this experiment may reinforce the possibility that studying brain integrative functions using USVs may be a useful tool for investigating the relationship between thalamic dysfunction and various psychiatric disorders, including schizophrenia, depression, and autism spectrum disorder [10,91].

Since locomotion and rearing were not affected after muscimol injection into the VPL, spontaneous behavior function appears to have been maintained. Furthermore, the absence or minimal change in the number of 50-kHz USVs, especially trill, complex, and step up FM, which are related to positive emotion and motivation [11,13,31], suggests that the rats’ emotional and motivational circuits were largely unaffected. By contrast, reward-seeking behaviors and exploring after rhythmic stroking, decreased after muscimol injection, indicating that the positive emotion (reward) circuit is maintained and motivation to seek reward is generated, but higher brain functions, comprising attention and cognition, which are required for object orientation and motor execution, are impaired. The notion is supported by the results of previous studies in which lesions of the RT, which primarily provides GABAergic input to the VPL [62], impair attentional orienting, which is essential to the execution of motivated behaviors [17,92,93]. In particular, since the VPL has no intrinsic GABAergic interneurons [63–65], the execution of motivated behaviors after rhythmic stroking is likely enabled through GABAergic neuron disinhibition in the RT that projects to the VPL.

Our recent study, which revealed that a lesion in the dorsal column largely decreased the number of 50-kHz USVs and motivated-exploratory behaviors, indicated that the dorsal column–medial lemniscus pathway, which projects to the VPL, may be involved in positive emotion induced by rhythmic stroking [12]. However, contrary to our prediction, we revealed that the VPL does not play a crucial role in producing 50-kHz USVs during and after tactile reward. The dorsal column tract projects to the DCN, which in turn projects to a wide range of nuclei in the brainstem and subcortical structures involved in emotion [48]. Specifically, the periaqueductal gray (PAG) is known to be involved in 50-kHz USV production induced by tickling, heterospecific play behavior, which requires external somatosensory stimulation, in juvenile rats [94]. Therefore, somatosensory information from rhythmic stroking may also activate the brain stem, such as PAG, without passing through the thalamic primary relay nuclei, thereby generating 50-kHz USVs.

Muscimol injection into the bilateral VPL totally impaired approach behavior, i.e., goal-directed reward-seeking behavior. The nucleus accumbens dopamine plays a crucial role in reward-directed approach [53] and 50-kHz USVs induced by rhythmic stroking [13]. In addition, it is reported that the ventral pallidum GABAergic neurons innervating the ventral tegmental area (VTA) strongly promoted arousal-related motivation via disinhibition of VTA dopaminergic neurons [95]. The present study reveals that GABAergic activation in the VPL impaired motivation system (reward-directed behavior) and spared emotion system (50-kHz USVs), indicating that the VPL GABAergic system may differentially regulate emotional and motivational integration, possibly via the basal ganglia including the nucleus accumbens and ventral pallidum innervated by the VTA. However, this idea remains speculative, and further investigation into the details of the integrative functions associated with emotion and motivation induced by external somatosensory stimuli is warranted.

Siviy and Panksepp [96] reported that the electrolytic lesion of the parafascicular nucleus of the thalamus and the posterior thalamic area reduced the con-specific play behavior while largely sparing motivation to play. However, destroying the ventral basal thalamus had only subtle effects on both measurements [96]. Burk and Mair [97] reported that intralaminar thalamic lesions differentially affect sensory attention and intentional motor function and suggested that the effects of intralaminar thalamic lesions on motor function were not related to sensory loss or attentional dysfunction. Considering the results of the present study and previous works, the thalamic nuclei may act as a gate integrating somatosensory input into motivation, emotion, and/or, attention through different mechanisms. This idea supports Wolff et al.’s theory that the thalamus appears as the bridge linking perception, cognition, and possibly affect [90].

Dividing 50-kHz subtypes into two and four types revealed slight differences in flat calls. Specifically, in the two-type classification method, there was an effect of muscimol administration of flat calls after stimulation. In contrast, in the four-type classification method, not only the effect of muscimol administration on flat calls during stimulation was confirmed but also on their occurrence rate. Flat calls may serve a socially coordinating function as a contact call [5,10,40,98]. Our previous study showed that flat calls were more likely to occur with low EEG theta activity and behavior [11], suggesting that muscimol administration may have caused an emotional and arousal state that affected the appearance of flat calls. These results clearly indicate the need to distinguish between flat and FM calls

Dividing 50-kHz USVs into two categories is useful because it simplifies analysis, given their minimal effects in this study. However, specific subtypes are strongly associated with specific behaviors during play anticipation [99,100] and may have different behavioral significance [101]. Burke and colleagues [102] have suggested that trill calls are related to the expectation of social interaction, i.e., motivated state. In our previous research using rhythmic stroking, completely different FM subtypes were observed during and after stimulation [11–13,28,31], suggesting a relationship between EEG and activity levels and specific USV subtypes [11]. Reportedly, the most common FM subtypes (trill, complex, harmonics, respectively) differ depending on somatosensory stimuli such as tickling, gentle back stroking, and rhythmic stroking [11,28,29,103], and this difference may be related to differences in arousal levels [11]. In addition, 50-kHz USVs are recorded even during nonrewarding situations [104,105]. Therefore, not adopting a detailed classification of FM may hinder the examination of the physiological significance of USVs. Since different FM calls are obtained depending on the stimuli, a detailed classification and examination of FM calls indicated that they can indicate integrative functions such as sensation, behavior, emotion, arousal, and cognition activated by stimulation. Depending on the experimental aims, it may be necessary to focus on a detailed classification of FM subtypes to apply USVs as behavioral markers for a wide range of brain integration functions.

In our laboratory, rats are placed close to a microphone and the sound during stimulation recorded at a nearly constant distance, so the sound intensity may affect the presence or absence of overtones. Since dB values in flat calls vary even within the same recording from the same individual (see S1 Fig), this study did not quantitatively compare dB values. However, since harmonics are clearly detectable even with lower dB sounds, it is unlikely that the harmonics observed in this study are artifacts caused by sound loudness. Both our current and past research consistently identified subtypes exhibiting harmonics during stimulation. Similar to the present results, several studies using different recording equipment have reported sonograms containing harmonic components [33,43,104]. If harmonics are a simple equipment artifact, it is difficult to explain why only the middle part of the split and part of the composite are harmonics. Research suggests that rats perceive octave equivalence, which may support the importance of the harmonic structure [105]. Accordingly, we believe that rats can produce harmonic vocalizations, and that “harmonic” vocalizations are not just noise but contain specific information.

In absence of harmonics, the sonogram shape is similar to the step FM [3], so flat harmonics may be simply a sound lacking the parts where the frequencies of other harmonics are modulated. Therefore, focusing only on sonogram parts without overtones, the harmonics type calls we consider special may be classified as step FM. Here, we classified them as per our previous research. It is beyond the scope of this study to clarify the meaning of overtones. As different classification methods yield different information, it is important to explore and improve the best classification method share as much as possible.

In this study, rats were group housed until immediately before the first day of handling and housed individually during handling and experimental periods. This rearing condition aimed to reduce the impact of the housing conditions on USVs. First, to avoid the effects on USVs difficult to control, such as aggression and defeater stress, which decrease positive 50-kHz USVs and produces negative 22-kHz USVs [5,106]. Second, to avoid the influence of isolation on USVs as much as possible. Specifically, socially isolation (~10 days) efficiently increases tickling-induced 50-kHz USVs, reflecting a positive affective state in juvenile rats [25,26,98] so socially isolated rats would be more motivated for tickling. Previously, we observed no changes in the number of USVs during rhythmic stroking in rats under three different isolation patterns: 0 days, 1 week, and 2 weeks [13,28,31]. Specifically, we previously showed that rhythmic stroking induced both abundant 50-kHz USVs and quick approach behavior even in naïve unhandled rats reared in groups until the day before the experiment [31]. Therefore, the experiment was conducted here such that the effects of group housing and the impact of individual rearing on USVs were either reduced or eliminated.

In this study, we used pentobarbital for initial anesthesia. Muscimol-stimulated chloride uptake in the cerebral cortex decreases following acute systemic high-dose (60 mg/kg) pentobarbital administration [107]. This uptake reduction decreases as the animal wakes up from anesthesia; the effect is reversible and short-lived. In this study, we used a low pentobarbital dose (40 mg/kg) and waited one week after surgery before performing the experiment, so it is unlikely that pentobarbital had any influence on the effects of muscimol. Moreover, the muscimol dose used in this study is typically used to inactivate brain nuclei in studies using pentobarbital for initial anesthesia [80,81].

## Limitations of the study

This study has several limitations. First, the extent of muscimol administration was confirmed only by observing the diffusion of EB under a light microscope; effects at the cellular level were not verified using immunohistochemical staining techniques. In addition, the potential impact of muscimol spread to adjacent regions such as VPM and RT, which also contain GABA receptors, was not investigated [62]. Thus, we cannot completely rule out the possibility that the GABAergic inhibition system of the RT and VPM may also be activated by muscimol in these animals where diffusion was confirmed. However, regardless of whether diffusion was present or not, muscimol administration consistently impaired approach behavior in all rats, in which the spread of EB in VPL was confirmed. Second, only a single dose of muscimol was studied, but the dose used in this study is commonly used in the thalamus and limbic system [80–83]. The thalamocortical relay neurons in the VPL are predominantly activated by driver inputs, which use ionotropic glutamate receptors, including AMPA and NMDA [54]. Further, the effects of lidocaine in decreasing input resistance and shunting action potentials in VPL thalamocortical neurons are not mediated by GABA_A_ receptors [108]. Therefore, GABA_A_ receptors may not be significantly involved in the sensory projection function from the thalamus representing the receptive field of the trunk to the cortex. In future studies, it will be important to confirm the involvement of the VPL by administering glutamate receptor blockers as a means of blocking tactile input to the VPL. Third, the use of only male rats in this study is an important limitation. As described in the Methods section, the present study used only male rats because it was designed to focus on the neural mechanisms of specific USVs that have been consistently observed in our series of studies in males [11–13,28,31]. Female rats have been shown to exhibit different vocal and behavioral responses to rewarding stimuli such as tickling [77,109,110] and these responses vary depending on the estrous cycle [111]. Incorporating both sexes is crucial for ensuring generalizability in biomedical research. Future studies including female rats will therefore be necessary to determine whether the findings of this study can be applied across sexes and to further investigate the influence of the estrous cycle and ovarian hormone levels on the mechanisms of somatosensory processing, cognition, emotion, and motivational behavior. Although this study has several limitations, its findings may help elucidate the ascending pathways that process positive emotions, which are still largely unknown [48]. In addition, the results of this study may support the idea that rat USVs can be a useful tool for elucidating the integrative functions of the brain [5,9,10].

## Conclusions

In conclusion, the VPL is essential for the execution of goal-directed reward-seeking behavior induced by tactile reward but plays a minor role in affective 50-kHz USVs. Our animal model using rhythmic stroking combined with USVs and motivated behaviors recording will be useful for investigating how somatosensory stimuli trigger emotions and motivations. Future studies are required to identify alternative neural pathways that do not involve the VPL GABA system as the neural pathways involved in positive 50-kHz USVs induced by somatosensory stimulation.

## Supporting information

S1 Fig Representative spectrograms of 50-kHz USVs during and after rhythmic stroking. (DOCX)

S2 Fig Guide cannula implantation surgery does not alter the number of 50-kHz USVs induced by rhythmic stroking (RS). (DOCX)

S3 Fig Muscimol injection into the VPL slightly alters two-category classification of 50-kHz USVs during and after rhythmic stroking. (DOCX)

S4 File Dataset for Figs 2–4. (XLSX)

S5 File Dataset for S2-3 Figs. (XLSX)
