# Supplementary material for: Muscimol injection into the ventral posterolateral nucleus of the thalamus impairs tactile reward-seeking behavior but preserves affective vocalization in male rats
Source: PLoS One. 2026 Jun 10;21(6):e0351495. doi: 10.1371/journal.pone.0351495 (PMC13252792; doi:10.1371/journal.pone.0351495)
Supplement: S1 Fig — (DOCX) [file pone.0351495.s001.docx]

A Flat calls during rhythmic stroking

Step up

Complex

Trill


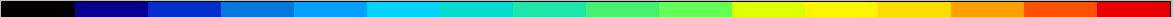


-50

-57

-61

-66

-71

-81

-76

-87

dB

Step up with harmonics

Step down with harmonics

Harmonic flat

Split


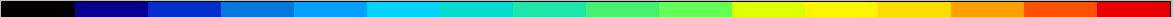


-42

-48

-53

-57

-62

-73

-67

-79

dB

B Harmonic calls during rhythmic stroking

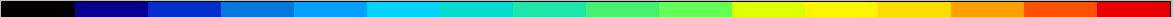


-42

-48

-53

-57

-62

-73

-67

-79

dB

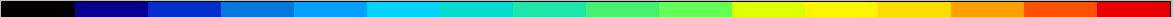


-42

-48

-53

-57

-62

-73

-67

-79

dB

C Trill + Complex + Step up after rhythmic stroking

**S1 Fig.**

**Representative spectrograms of 50-kHz USVs during and after rhythmic stroking.**

Spectrograms were recorded during and after rhythmic stroking prior to vehicle injection in a single rat. Left, amplitude spectrum of the call (V); right, spectrogram with time (ms/s) on the horizontal axis and frequency (kHz) on the vertical axis. Colors indicate the relative signal intensity (dB).
