# Supplementary material for: Muscimol injection into the ventral posterolateral nucleus of the thalamus impairs tactile reward-seeking behavior but preserves affective vocalization in male rats
Source: PLoS One. 2026 Jun 10;21(6):e0351495. doi: 10.1371/journal.pone.0351495 (PMC13252792; doi:10.1371/journal.pone.0351495)
Supplement: S2 Fig — (DOCX) [file pone.0351495.s002.docx]

**
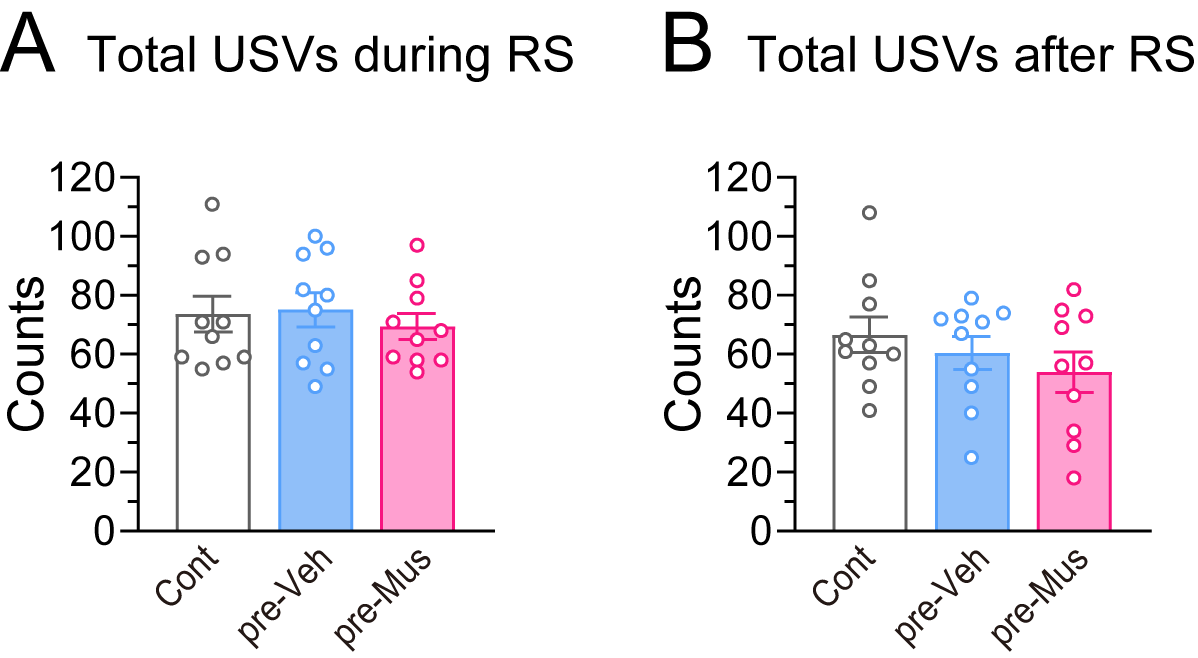
**

**S2 Fig.**

**Guide cannula implantation surgery does not alter the number of 50-kHz USVs induced by rhythmic stroking (RS).** Shown are the total number of 50-kHz USVs recorded during (A) and after (B) rhythmic stroking in rats before surgery (Cont) and prior to vehicle (pre-Veh) or muscimol (pre-Mus) injection after surgery. Group means were compared by RM-ANOVA. N = 10.
