# Supplementary material for: Muscimol injection into the ventral posterolateral nucleus of the thalamus impairs tactile reward-seeking behavior but preserves affective vocalization in male rats
Source: PLoS One. 2026 Jun 10;21(6):e0351495. doi: 10.1371/journal.pone.0351495 (PMC13252792; doi:10.1371/journal.pone.0351495)
Supplement: S3 Fig — (DOCX) [file pone.0351495.s003.docx]

**
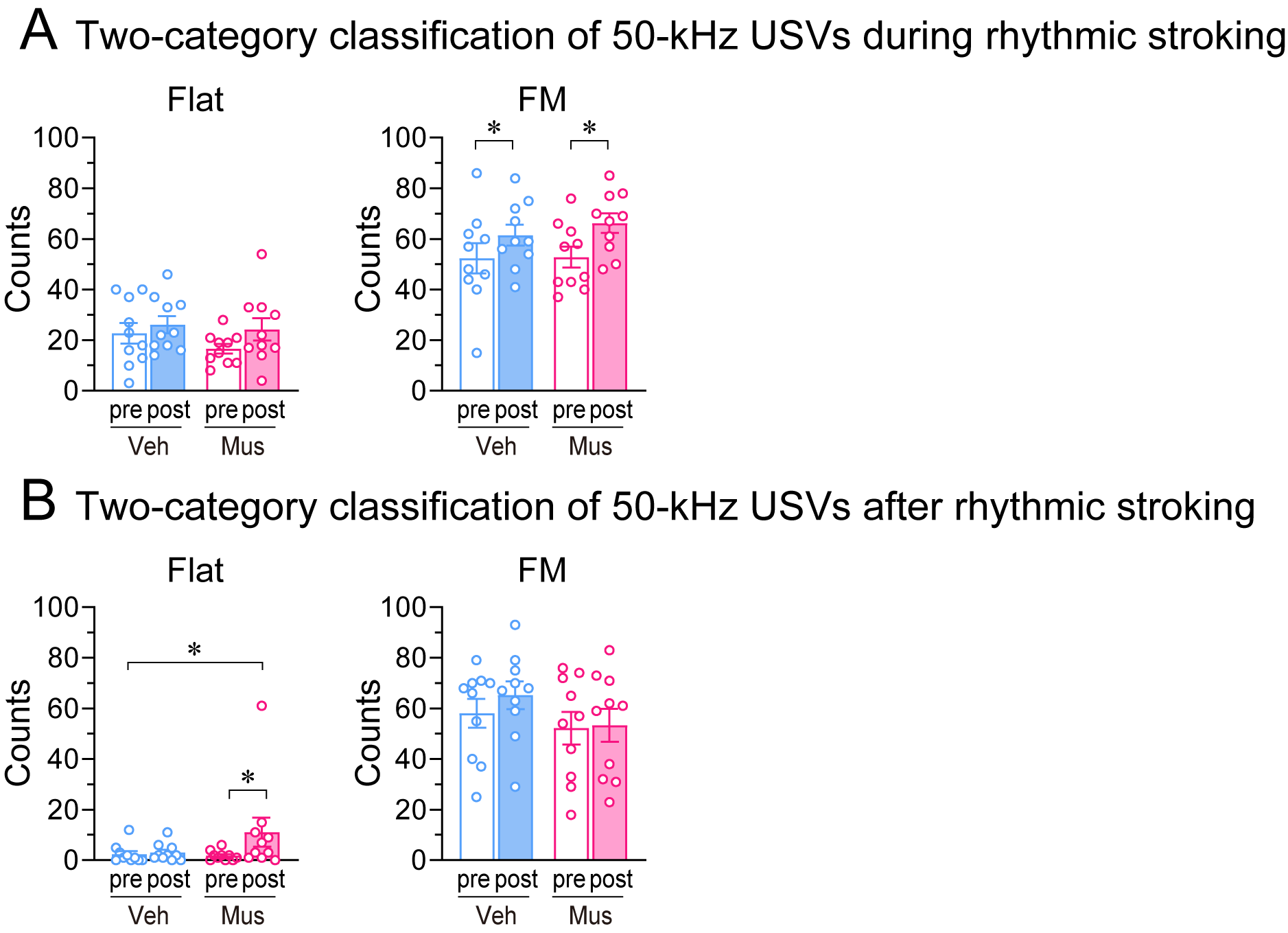
**

**S3 Fig.**

**Muscimol injection into the VPL slightly alters two-category classification of 50-kHz USVs during and after rhythmic stroking.**

Number of flat and FM calls during (A) and after (B) rhythmic stroking in rats before and after vehicle (Veh) or muscimol (Mus) injection. * p < 0.05 compared with pre-vehicle or pre-muscimol injection using the paired t-test or Wilcoxon signed-rank test. N = 10.
