# Supplementary material for: Muscimol injection into the ventral posterolateral nucleus of the thalamus impairs tactile reward-seeking behavior but preserves affective vocalization in male rats
Source: PLoS One. 2026 Jun 10;21(6):e0351495. doi: 10.1371/journal.pone.0351495 (PMC13252792; doi:10.1371/journal.pone.0351495)
Supplement: Attachment — Submitted filename: Response to Reviewers.docx [file pone.0351495.s007.docx]

**Responses to the Editor**

We gratefully acknowledge the Editor, Reviewers, and Journal Officer for their insightful feedback and constructive comments on our manuscript, which have significantly enhanced its quality. Please find below our responses to the journal’s requirements.

Journal Requirements:

When submitting your revision, we need you to address these additional requirements.

1. Please ensure that your manuscript meets PLOS ONE's style requirements, including those for file naming. The PLOS ONE style templates can be found at

https://journals.plos.org/plosone/s/file?id=wjVg/PLOSOne_formatting_sample_main_body.pdf and

https://journals.plos.org/plosone/s/file?id=ba62/PLOSOne_formatting_sample_title_authors_affiliations.pdf

**Response:**

We revised the text and can confirm that it follows the style requirements of *PLOS ONE*.

2. We note that the grant information you provided in the ‘Funding Information’ and ‘Financial Disclosure’ sections do not match.

When you resubmit, please ensure that you provide the correct grant numbers for the awards you received for your study in the ‘Funding Information’ section.

**Response:**

Thank you for identifying this issue. We corrected the grant information accordingly.

3. We note that Figure 1 in your submission contain copyrighted images. All PLOS content is published under the Creative Commons Attribution License (CC BY 4.0), which means that the manuscript, images, and Supporting Information files will be freely available online, and any third party is permitted to access, download, copy, distribute, and use these materials in any way, even commercially, with proper attribution. For more information, see our copyright guidelines: http://journals.plos.org/plosone/s/licenses-and-copyright.

We require you to either (1) present written permission from the copyright holder to publish these figures specifically under the CC BY 4.0 license, or (2) remove the figures from your submission:

1. You may seek permission from the original copyright holder of Figure 1 to publish the content specifically under the CC BY 4.0 license.

**Response:**

We appreciate your remarks. We have removed the figures (Fig. 1C) and accompanying text because we were unable to obtain permission from the original copyright holders to publish these figures under the CC BY 4.0 license.

4. If the reviewer comments include a recommendation to cite specific previously published works, please review and evaluate these publications to determine whether they are relevant and should be cited. There is no requirement to cite these works unless the editor has indicated otherwise.

**Response:**

Thank you for this clarification. We carefully reviewed the publications suggested in the reviewer comments and evaluated their relevance to the scope and context of our study. Where appropriate and relevant to the discussion of ultrasonic vocalizations and their interpretation, we have incorporated the recommended references and discussed them in the manuscript. We also ensured that the cited literature reflects the broader body of work in this field while maintaining the focus of the present study.

**Response to Reviewer #1**

Thank you for your valuable comments on our manuscript. We have revised the manuscript accordingly. Our responses to your comments are provided below.

**Animals:** please provide a clear description of the experimental groups. It is stated that 10 animals were used and that 4 animals were used per experiment. Please indicate how many experiments were performed, if rats were re-used and how. Moreover, it is reported that rats were individually housed. Since isolation can alter some dimension of ultrasonic vocalization in rats (see for example, 10.1016/j.bbr.2022.114157), it should be discussed whether and how individual housing may have influenced the results obtained.

**Response:**

Thank you for your comments. We corrected the number of rats used and added a discussion about the influence of individual housing (page 10, line 154; page 33–34, lines 561–576). A total of 12 rats were used in three experiments with four rats in each. Of all rats, two were used in a preliminary experiment, and excluded from data analysis and the remaining 10 were used. The original manuscript indicated four animals; we revised the text to indicate that there were 2–4 animals, excluding the two used in the preliminary experiment.

**Surgery:** please discuss whether the use of pentobarbital, which targets GABAergic receptors, may be expected to have any residual influences on the effects of muscimol evaluated at a later point. Moreover, please indicate which antibiotic therapy was used.

**Response:**

We revised the discussion accordingly (page 34, lines 577–584). The antibiotic therapy used in this study was specified in the text (page 11, lines 179–180).

Microinjection of GABA-A receptor agonists: “agonists” should be in singular form, since only muscimol was used. Moreover, the use of a single dose of muscimol should be acknowledged as a potential limitation of the present study.

**Response:**

Thank you for your comments. We revised the text accordingly and included the use of a single dose of muscimol as a limitation (page 11, line 164; page 12, line 183; pages 34–35, lines 591–601).

Recording and analysis of ultrasonic vocalizations: “Specifically, trill, complex, and step up calls, which have been shown to be mediated by the accumbal dopamine system [10] are among the frequency-modulated (FM) calls that are indicators of positive emotions”. This sentence contains a series of overstatements. First, the quoted review article does not say that. Second trill, complex and step up calls are not the only to be mediated by accumbal dopamine system, since all the 50 kHz ultrasonic vocalizations depend on the accumbal dopamine system (see, for example: 10.1016/j.conb.2013.01.014; 10.1016/j.jneumeth.2018.06.018). Third, the use of those specific subtypes of ultrasonic vocalizations as indicators of positive emotions is a popular approach in the ultrasonic vocalization community, that however still lacks a final validation. Proof of this is the evidence from several studies by different groups that rats may emit “flat” 50 kHz ultrasonic vocalizations in situations that are believed to have positive emotional valence (see for example, 0.1016/j.pnpbp.2018.07.002; 10.1016/j.bbr.2018.02.041). Accordingly, the author must tone down these statements and discuss them in the framework of the existing literature on ultrasonic vocalizations. If the author wish to focus on the specific call subtypes reported, this may be fine, but the significance of those calls should be presented in a fair way, with reference to the existing literature, that must be acknowledged and discussed, and with reference to the earlier studies by the same author that investigated those specific call subtypes [i.e., references 10 and 12 quoted in the present manuscript).

**Response:**

Thank you for your insightful comments. We agree, so we corrected the text accordingly (page 13 line 206; page 20 line 337; page 27 lines 459–460).

Histological verification of injection sites: please clarify if the rats displaying a spread of dye to the VPM or RT were included in the study or excluded from the study.

**Response:**

Thank you for your comments. We revised the text accordingly (page 17, lines 286–288).

Bilateral muscimol injection into the VPL preserves affective 50-kHz USVs during and after rhythmic stroking: this paragraph and the associated figure must be reorganized. Again, the categorization of FM calls in “hedonic” and “other” is, at best, arbitrary. Again, no evidence exists to conclude that certain types of FM 50 kHz ultrasonic vocalizations communicate the presence of positive affect better than others, This is believed to occur for trill calls, but not all the available pieces of experimental evidence agree on this. Plus, on which basis were “split” calls dealt with as a separate category? Moreover, please define “harmonics”. Consider that according to some manufacturers of ultrasonic recording equipment (for example, Noldus) “harmonics” do not even exist as a real entity, rather being an artifact of the recording system used. Furthermore, this categorization in “hedonic” and “other” FM calls is also challenged by the very same results of this study that found an increase in flat calls. All this said, it is recommended that a new categorization of calls in flat and FM is used and presented, since the categorization proposed here may be criticized in many instances.

**Response:**

Thank you for your insightful and valuable comments. We revised the text accordingly (pages 3–5, lines 20–63; page 10, lines 149–152; page 13, lines 205–216; pages 20–21, lines 342–357; page 22, lines 372–374, pages 22–23, 378–380; page 23, line 389; pages 30–33, lines 519–560). We agree on the lack of evidence to conclude that any specific subtype of 50 kHz USV conveys a positive emotional state better than other vocalizations. According to your comments, we present the results in two categories: FM and Flat with a focus on the specific call subtypes we reported and their underlying mechanisms. Based on the points you raised, we improved our classification method, unifying split and harmonics rather than separating them, and presented four categories. In addition, we referred to existing literature, recognized and discussed it, and referred to our previous studies investigating those specific call subtypes, ensuring that we do not deviate from the existing framework.

Discussion: “The results of this experiment may support the idea that thalamic dysfunction is linked to various psychiatric disorders, including schizophrenia, major depressive disorder, and autism spectrum disorder [53]”. Again, this sentence is an overstatement that is based on the hypothesis that 50 kHz ultrasonic vocalizations reflect positive affect only, which may be not always the case (see, for example: 10.1016/j.neubiorev.2023.105260; 10.1007/s40732-025-00633-4; 10.1016/j.pnpbp.2020.110184). Therefore, the author must also present their data in the framework of the current evidence on the behavioral significance of ultrasonic vocalizations broadly speaking, and considering them not simply as indicators of positive affect. The same considerations apply to the paragraph “Conclusions”, where the last sentence must be toned down.

**Response:**

Thank you for your valuable comments. We revised the text accordingly (page 26, lines 450–456; page 36, lines 618–620).

Discussion: “Furthermore, the absence or minimal change in the number of 50-kHz USVs, especially hedonic FM, which serves as an index of positive emotion and motivation [10, 11, 48], suggests that the rats’ emotional and motivational circuits were largely unaffected”. Again, this sentence is an overstatement that must be toned down. Please see my previous comments.

**Response:**

Thank you for your valuable comments. We revised the text accordingly (page 27, lines 459–460).

**Response to Reviewer #2**

Thank you for your valuable comments on our manuscript. We have revised the manuscript accordingly. Our responses to your comments are provided below.

1. The manuscript lacks a clearly scientific rationale.

**Response:**

We appreciate your point regarding the scientific rationale. We included background information about the use of ultrasonic vocalizations in the Introduction to strengthen the basis of this experiment (page 2, lines 2–3; pages 3–5, lines 20–63).

2. This study is largely descriptive. Incorporating mechanistic explanations/experiments would substantially strengthen the conclusions.

**Response:**

Thank you for your comments. We revised the text accordingly (pages 30–33, lines 519–560; pages 34–35, lines 586–611).

3. Substantial information is missing in the section of Methods. For example, the rationale for including only male rats should be clearly explained, given the potential sex differences in affective vocalization and reward-related behaviors.

**Response:**

Thank you for your comments. We revised the text accordingly **(**page 10, lines 149–152; page 35–36, lines 609–619).

4. More information is needed regarding the anatomical specificity of the muscimol injection. Please clarify how the drug diffusion was restricted to the VPL and whether adjacent thalamic nuclei might have been affected.

**Response:**

Thank you for your comments. We revised the text accordingly (page 7, lines 287–288; page 35, lines 601–611).

5. This manuscript lacks in-depth discussion. It is highly recommended to provide more comprehensive discussions, take-home messages, etc.

Response:

Thank you for your valuable comments. We revised the text accordingly (page 26–27, lines 450–456; page 36, lines 618–620). We agree that a deeper interpretation of our findings would strengthen the manuscript. Accordingly, we thoroughly revised the Discussion to provide a more in-depth analysis of the implications of our results. Specifically, we expanded on the mechanistic insights of our results and included a Conclusion that highlights the practical significance of our findings for the field. We believe these additional address the lack of depth and enhance the contribution of our work.
