# Supplementary material for: Muscimol injection into the ventral posterolateral nucleus of the thalamus impairs tactile reward-seeking behavior but preserves affective vocalization in male rats
Source: PLoS One. 2026 Jun 10;21(6):e0351495. doi: 10.1371/journal.pone.0351495 (PMC13252792; doi:10.1371/journal.pone.0351495)
Supplement: Attachment — Submitted filename: Response to Reviewer.docx [file pone.0351495.s008.docx]

**Response to Editor**

We appreciate the editor and reviewers for their valuable comments regarding our manuscript and have made revisions accordingly. The following are the responses to the editor’s comments. Revisions in the revised manuscript are in red font.

**For specific Comments**

1. Please note that Reviewer 1 points out that it is absolutely necessary to describe the sample size adequately, and that a sample size of 4 is not sufficient for the experiments performed.

**Response:**

We sincerely apologize for the misleading wording. We corrected the text to indicate that the number of samples used in the experiment was 10 (page 10–11, lines 156–165). Furthermore, as stated on page 15 (line 247), the study was conducted using a within-subject design; thus, all 10 rats received the vehicle and muscimol.

2. Upon request, Reviewer #2 provided more clarification to his: Review Comments to the Author":

For Question 1, a clear scientific rationale should be provided rather than listing background information.

For Question 3, a justification for including only male subjects is required, rather than a descriptive statement.

For Question 4, the authors state: “Although slight diffusion was observed in some rats, all 10 rats were used in the data because the bilateral cannula tips were almost located in the target position.” It is unclear what is meant by “slight diffusion” and “almost”. What methods were used to assess or validate this? In addition, the histological results appear to show substantial tissue damage along the track.

Please address these points clearly in your response.

**Response:**

Thank you for confirming that we could obtain more detailed information regarding reviewer #2's comments. We addressed each of your points in the response to reviewer #2.

(Questions 1: page 9, lines 136–139; Question 3: page 10, lines 152–156; page 36, lines 616–623; Question 4: page 35, lines 594–598, lines 602–603).

Journal Requirements:

If the reviewer comments include a recommendation to cite specific previously published works, please review and evaluate these publications to determine whether they are relevant and should be cited. There is no requirement to cite these works unless the editor has indicated otherwise.

**Response:**

Thank you for your explanation. We have carefully considered the comments provided by Reviewer #2 and evaluated their relevance to the scope and context of this study. In response, we have cited and incorporated literature that is both appropriate and pertinent to justifying the exclusive use of male rats. We have also ensured that the cited references represent a broad range of research findings in this field, while maintaining the specific focus of the present study.

**Response to Reviewer #1**

Thank you for your valuable comments. We have revised the manuscript accordingly, and our responses are provided below.

The author has addressed most of my comments, but a critical issue remains, which concerns the sample size for each experiment. Specifically: figure legends report that a total of 10 animals were used but do not report how many animals were used for each evaluation. The author replied that "A total of 12 rats were used in three experiments with four rats in each. Of all rats, two were used in a preliminary experiment, and excluded from data analysis and the remaining 10 were used. The original manuscript indicated four animals; we revised the text to indicate that there were 2–4 animals, excluding the two used in the preliminary experiment". However, it is still unclear how may animals were used in each evaluation. This must be clearly specified. Please note that a sample size of 4 would be too low, given the variability in ultrasonic vocalization among rats, and would dramatically reduce the robustness of data.

**Response:**

We would like to thank Reviewer #1 for indicating that our previous response did not fully clarify the sample size (n = 10). We apologize for the lack of clarity in the previous version and for not adequately addressing this point. To respond more appropriately, we have revised the text accordingly (page 10–11, lines 156–165).

**Response to Reviewer #2**

Thank you for your valuable comments. We have revised the manuscript accordingly and our responses are provided below.

**Major comments**

The author did not fully address my previous question. There is no clear improvement in the revised version.

**Response:**

We deeply appreciate your time in reviewing our manuscript. We apologize for not being able to adequately address your requests for feedback. Regarding the portions of Reviewer #2’s previous questions that we did not clearly answer, we provide our responses to the specific comments below that were received from the editor.

**Specific comments**

1. For Question 1, a clear scientific rationale should be provided rather than listing background information.

**Response:**

We thank Reviewer #2 for taking the time to review our manuscript. To add a clear scientific rationale, we added a section on novelty, knowledge gaps, and the significance of the study to the final paragraph of the Introduction (page 9, lines 136–139). We hope that our understanding and this revision satisfy Reviewer #2’s points. If the revision is not consistent with Reviewer #2's views, we would appreciate additional feedback. Reviewer #2 indicated that we need “scientific rationale” rather than just a list of references. Does this mean reducing the background explanation or focusing directly on the research topic? We would like to confirm whether the revisions meet your expectations. Notably, when using rat ultrasonic vocalizations as a behavioral indicator, as in this study, it is necessary to explain to the reader that rat ultrasonic vocalizations are an indicator of positive emotions, that there are problems with the classification method of ultrasonic vocalizations (within the scope of Reviewer #1’s points), that rhythmic stroking is a stimulation method unique to our laboratory, and that we are studying a niche topic, which is the effect of somatosensory stimulation on emotion. This inevitably makes the background explanation lengthy. We would appreciate it if more specific feedback could be provided on the lack of scientific rationale as we are prepared to make revisions accordingly.

2. For Question 3, a justification for including only male subjects is required, rather than a descriptive statement.

**Response:**

Thank you for your valuable comments. We have revised the text to justify the use of only male rats (page 10, lines 152–156; page 36, lines 616–623).

3. For Question 4, the authors state: “Although slight diffusion was observed in some rats, all 10 rats were used in the data because the bilateral cannula tips were almost located in the target position.” It is unclear what is meant by “slight diffusion” and “almost”. What methods were used to assess or validate this? In addition, the histological results appear to show substantial tissue damage along the track.

**Response:**

Thank you for your valuable comments. In this study, we examined the localization of Evans Blue (EB) administration to the VPL by observing its diffusion under a light microscope. We acknowledge the limitation that cellular-level effects were not assessed using immunohistochemical techniques (page 35, lines 594–598). In addition, we have removed sections that provided vague descriptions of EB diffusion, as this was not the primary focus of the study (page 18, lines 288–292).

In the microinjection experiment, the degree of tissue damage caused by guide cannula placements is usually present to the extent observed in this study. However, we felt that it was necessary to show that there was no significant effect on the USV before and after tissue damage, and therefore, we added a supplementary fig. S2 (page 20, lines 331–335).
