# Supplementary material for: Muscimol injection into the ventral posterolateral nucleus of the thalamus impairs tactile reward-seeking behavior but preserves affective vocalization in male rats
Source: PLoS One. 2026 Jun 10;21(6):e0351495. doi: 10.1371/journal.pone.0351495 (PMC13252792; doi:10.1371/journal.pone.0351495)
Supplement: Attachment — Submitted filename: Response_to_Reviewers_auresp_3.docx [file pone.0351495.s009.docx]

**Response to Editor:**

We appreciate the editor and reviewers for their valuable comments regarding our manuscript. We have revised the manuscript accordingly. Following are the responses to the editor’s comments. Please note that the revisions are indicated in red font.

**ACADEMIC EDITOR:**

I agree with reviewer #2 that since only male animals were used, the title should be changed to contain the word 'male' ('....but preserves affective vocalizations in rats'). If the author has a specific justification for only using male mice, this could be added.

**Response:**

Thank you for your valuable comments. We agree that it should be clearly stated to the readers that only male rats were used in study. Accordingly, we have updated the title to “Muscimol injection into the ventral posterolateral nucleus of the thalamus impairs tactile reward-seeking behavior but preserves affective vocalization in male rats.” The reason for using only male animals is that the effects of changes in psychosomatic functions associated with fluctuations in the estrous cycle of female rats on emotions during tactile stimulation are still unknown, and thus, experiments using female rats would be beyond the scope of this research topic. This point has already been described in the methods and research limitations sections (page 10, lines 152–156; page 36, lines 612–623).

Journal Requirements:

1. If the reviewer comments include a recommendation to cite specific previously published works, please review and evaluate these publications to determine whether they are relevant and should be cited. There is no requirement to cite these works unless the editor has indicated otherwise.

**Response:**

Thank you for your valuable comment. We have carefully considered the comments provided by Reviewer #2 and evaluated their relevance to the scope and context of this study. The reviewers’ comments did not include any recommendations to cite specific previously published literature, and therefore, I did not cite any literature separately.

2. Please review your reference list to ensure that it is complete and correct. If you have cited papers that have been retracted, please include the rationale for doing so in the manuscript text, or remove these references and replace them with relevant current references. Any changes to the reference list should be mentioned in the rebuttal letter that accompanies your revised manuscript. If you need to cite a retracted article, indicate the article’s retracted status in the References list and also include a citation and full reference for the retraction notice.

**Response:**

We appreciate your insightful comments. We have confirmed that no retracted articles are included and that all references are complete and accurate.

**Response to Reviewer #1**

Thank you for your valuable comments.

**Major comments**

The author has addressed all my concerns. The description of experimental groups is now clear. I have no further comments.

**Response:**

Thank you for taking the time to review our manuscript, providing constructive feedback, and conducting a thorough review of the manuscript. We are also grateful for your assessment that we were able to adequately address the points you raised in the revisions.

**Response to Reviewer #2**

Thank you for your valuable comments. We have revised the manuscript accordingly and our responses are provided below.

**Specific comments**

there is still no explanation for why only male subjects were used. I would recommend adding “male” to the title to avoid misleading the audience.

**Response:**

We are grateful to you for this constructive feedback and for the specific guidance. We agree that it should be clearly communicated to readers that only male rats were used. As such, we have updated the title to “Muscimol injection into the ventral posterolateral nucleus of the thalamus impairs tactile reward-seeking behavior but preserves affective vocalization in male rats.” We sincerely apologize that we were unable to provide a response that met your expectations. Although we have stated our reasoning and research limitations (page 10, lines 152–156; page 36, lines 612–623), we would be extremely grateful for any further guidance you may have. Thank you very much for taking the time to review our paper.
