# Supplementary material for: Muscimol injection into the ventral posterolateral nucleus of the thalamus impairs tactile reward-seeking behavior but preserves affective vocalization in male rats
Source: PLoS One. 2026 Jun 10;21(6):e0351495. doi: 10.1371/journal.pone.0351495 (PMC13252792; doi:10.1371/journal.pone.0351495)
Supplement: Peer review history

# Peer review: Decision Letter 0

**DOI:** 10.1371/journal.pone.0351495.r001

**Authors:** Uwe Rudolph

24 Feb 2026

PONE-D-26-02461

Muscimol injection into the ventral posterolateral nucleus of the thalamus impairs tactile reward-seeking behavior but preserves affective vocalization in rats

PLOS One

Dear Dr. Shimoju,

Thank you for submitting your manuscript to PLOS ONE. After careful consideration, we feel that it has merit but does not fully meet PLOS ONE’s publication criteria as it currently stands. Both reviewers raise multiple points that need to be addressed in a revised version.

Therefore, we invite you to submit a revised version of the manuscript that addresses the points raised during the review process. Please include a pointy-by-point response to the reviewers' concerns.

Please submit your revised manuscript by Apr 10 2026 11:59PM. If you will need more time than this to complete your revisions, please reply to this message or contact the journal office at plosone@plos.org. When you're ready to submit your revision, log on to https://www.editorialmanager.com/pone/ and select the 'Submissions Needing Revision' folder to locate your manuscript file.

Please include the following items when submitting your revised manuscript:

- A letter that responds to each point raised by the academic editor and reviewer(s). You should upload this letter as a separate file labeled 'Response to Reviewers'.
- A marked-up copy of your manuscript that highlights changes made to the original version. You should upload this as a separate file labeled 'Revised Manuscript with Track Changes'.
- An unmarked version of your revised paper without tracked changes. You should upload this as a separate file labeled 'Manuscript'.

If you would like to make changes to your financial disclosure, please include your updated statement in your cover letter. Guidelines for resubmitting your figure files are available below the reviewer comments at the end of this letter.

If applicable, we recommend that you deposit your laboratory protocols in protocols.io to enhance the reproducibility of your results. Protocols.io assigns your protocol its own identifier (DOI) so that it can be cited independently in the future. For instructions see: https://journals.plos.org/plosone/s/submission-guidelines#loc-laboratory-protocols. Additionally, PLOS ONE offers an option for publishing peer-reviewed Lab Protocol articles, which describe protocols hosted on protocols.io. Read more information on sharing protocols at https://plos.org/protocols?utm_medium=editorial-email&utm_source=authorletters&utm_campaign=protocols.

We look forward to receiving your revised manuscript.

Kind regards,

Uwe Rudolph

Academic Editor

PLOS One

Journal Requirements:

When submitting your revision, we need you to address these additional requirements.

1. Please ensure that your manuscript meets PLOS ONE's style requirements, including those for file naming. The PLOS ONE style templates can be found at

https://journals.plos.org/plosone/s/file?id=wjVg/PLOSOne_formatting_sample_main_body.pdf and

https://journals.plos.org/plosone/s/file?id=ba62/PLOSOne_formatting_sample_title_authors_affiliations.pdf

2.  We note that the grant information you provided in the ‘Funding Information’ and ‘Financial Disclosure’ sections do not match.

When you resubmit, please ensure that you provide the correct grant numbers for the awards you received for your study in the ‘Funding Information’ section.

3. We note that Figure 1 in your submission contain copyrighted images. All PLOS content is published under the Creative Commons Attribution License (CC BY 4.0), which means that the manuscript, images, and Supporting Information files will be freely available online, and any third party is permitted to access, download, copy, distribute, and use these materials in any way, even commercially, with proper attribution. For more information, see our copyright guidelines: http://journals.plos.org/plosone/s/licenses-and-copyright.

We require you to either (1) present written permission from the copyright holder to publish these figures specifically under the CC BY 4.0 license, or (2) remove the figures from your submission:

1. You may seek permission from the original copyright holder of Figure 1 to publish the content specifically under the CC BY 4.0 license.

We recommend that you contact the original copyright holder with the Content Permission Form (http://journals.plos.org/plosone/s/file?id=7c09/content-permission-form.pdf) and the following text:

“I request permission for the open-access journal PLOS ONE to publish XXX under the Creative Commons Attribution License (CCAL) CC BY 4.0 (http://creativecommons.org/licenses/by/4.0/). Please be aware that this license allows unrestricted use and distribution, even commercially, by third parties. Please reply and provide explicit written permission to publish XXX under a CC BY license and complete the attached form.”

Please upload the completed Content Permission Form or other proof of granted permissions as an "Other" file with your submission.

In the figure caption of the copyrighted figure, please include the following text: “Reprinted from [ref] under a CC BY license, with permission from [name of publisher], original copyright [original copyright year].”

2. If you are unable to obtain permission from the original copyright holder to publish these figures under the CC BY 4.0 license or if the copyright holder’s requirements are incompatible with the CC BY 4.0 license, please either i) remove the figure or ii) supply a replacement figure that complies with the CC BY 4.0 license. Please check copyright information on all replacement figures and update the figure caption with source information. If applicable, please specify in the figure caption text when a figure is similar but not identical to the original image and is therefore for illustrative purposes only.

4. If the reviewer comments include a recommendation to cite specific previously published works, please review and evaluate these publications to determine whether they are relevant and should be cited. There is no requirement to cite these works unless the editor has indicated otherwise.

[Note: HTML markup is below. Please do not edit.]

Reviewers' comments:

Reviewer's Responses to Questions

**Comments to the Author**

1. Is the manuscript technically sound, and do the data support the conclusions?

The manuscript must describe a technically sound piece of scientific research with data that supports the conclusions. Experiments must have been conducted rigorously, with appropriate controls, replication, and sample sizes. The conclusions must be drawn appropriately based on the data presented.

Reviewer #1: Partly

Reviewer #2: Yes

**********

2. Has the statistical analysis been performed appropriately and rigorously?

Reviewer #1: Yes

Reviewer #2: Yes

**********

3. Have the authors made all data underlying the findings in their manuscript fully available?

The PLOS Data policy requires authors to make all data underlying the findings described in their manuscript fully available without restriction, with rare exception (please refer to the Data Availability Statement in the manuscript PDF file). The data should be provided as part of the manuscript or its supporting information, or deposited to a public repository. For example, in addition to summary statistics, the data points behind means, medians and variance measures should be available. If there are restrictions on publicly sharing data—e.g. participant privacy or use of data from a third party—those must be specified.

Reviewer #1: Yes

Reviewer #2: Yes

**********

4. Is the manuscript presented in an intelligible fashion and written in standard English?

PLOS ONE does not copyedit accepted manuscripts, so the language in submitted articles must be clear, correct, and unambiguous. Any typographical or grammatical errors should be corrected at revision, so please note any specific errors here.

Reviewer #1: Yes

Reviewer #2: Yes

**********

5. Review Comments to the Author

Please use the space provided to explain your answers to the questions above. You may also include additional comments for the author, including concerns about dual publication, research ethics, or publication ethics. (Please upload your review as an attachment if it exceeds 20,000 characters)

Reviewer #1: The present study evaluated the effects of the injection of muscimol into the ventral posterolateral nucleus of the thalamus on ultrasonic vocalization and approach behavior stimulated by gentle stroking in rats. The results obtained indicated that muscimol marginally affected the emission of 50 kHz ultrasonic vocalizations, while significantly impacting approach behavior. The results are discussed in the framework of motivation, reward and neurocircuits involved.

While the present results may be of interest, there are several parts of the present study that require clarification. In particular, the author must pay great attention to the classification of 50 kHz ultrasonic vocalization simply as an indicator of positive affect and to the call categorization used. Furthermore, the manuscript contains several overstatements that must be toned down, and discussed in the framework of the existing literature ion the field.

Please find my detailed comments below.

Animals: please provide a clear description of the experimental groups. It is stated that 10 animals were used and that 4 animals were used per experiment. Please indicate how many experiments were performed, if rats were re-used and how. Moreover, it is reported that rats were individually housed. Since isolation can alter some dimension of ultrasonic vocalization in rats (see for example, 10.1016/j.bbr.2022.114157), it should be discussed whether and how individual housing may have influenced the results obtained.

Surgery: please discuss whether the use of pentobarbital, which targets GABAergic receptors, may be expected to have any residual influences on the effects of muscimol evaluated at a later point. Moreover, please indicate which antibiotic therapy was used.

Microinjection of GABA-A receptor agonists: “agonists” should be in singular form, since only muscimol was used. Moreover, the use of a single dose of muscimol should be acknowledged as a potential limitation of the present study.

Recording and analysis of ultrasonic vocalizations: “Specifically, trill, complex, and step up calls, which have been shown to be mediated by the accumbal dopamine system [10] are among the frequency-modulated (FM) calls that are indicators of positive emotions”. This sentence contains a series of overstatements. First, the quoted review article does not say that. Second trill, complex and step up calls are not the only to be mediated by accumbal dopamine system, since all the 50 kHz ultrasonic vocalizations depend on the accumbal dopamine system (see, for example: 10.1016/j.conb.2013.01.014; 10.1016/j.jneumeth.2018.06.018). Third, the use of those specific subtypes of ultrasonic vocalizations as indicators of positive emotions is a popular approach in the ultrasonic vocalization community, that however still lacks a final validation. Proof of this is the evidence from several studies by different groups that rats may emit “flat” 50 kHz ultrasonic vocalizations in situations that are believed to have positive emotional valence (see for example, 0.1016/j.pnpbp.2018.07.002; 10.1016/j.bbr.2018.02.041). Accordingly, the author must tone down these statements and discuss them in the framework of the existing literature on ultrasonic vocalizations. If the author wish to focus on the specific call subtypes reported, this may be fine, but the significance of those calls should be presented in a fair way, with reference to the existing literature, that must be acknowledged and discussed, and with reference to the earlier studies by the same author that investigated those specific call subtypes [i.e., references 10 and 12 quoted in the present manuscript).

Histological verification of injection sites: please clarify if the rats displaying a spread of dye to the VPM or RT were included in the study or excluded from the study.

Bilateral muscimol injection into the VPL preserves affective 50-kHz USVs during and after rhythmic stroking: this paragraph and the associated figure must be reorganized. Again, the categorization of FM calls in “hedonic” and “other” is, at best, arbitrary. Again, no evidence exists to conclude that certain types of FM 50 kHz ultrasonic vocalizations communicate the presence of positive affect better than others, This is believed to occur for trill calls, but not all the available pieces of experimental evidence agree on this. Plus, on which basis were “split” calls dealt with as a separate category? Moreover, please define “harmonics”. Consider that according to some manufacturers of ultrasonic recording equipment (for example, Noldus) “harmonics” do not even exist as a real entity, rather being an artifact of the recording system used. Furthermore, this categorization in “hedonic” and “other” FM calls is also challenged by the very same results of this study that found an increase in flat calls. All this said, it is recommended that a new categorization of calls in flat and FM is used and presented, since the categorization proposed here may be criticized in many instances.

Discussion: “The results of this experiment may support the idea that thalamic dysfunction is linked to various psychiatric disorders, including schizophrenia, major depressive disorder, and autism spectrum disorder [53]”. Again, this sentence is an overstatement that is based on the hypothesis that 50 kHz ultrasonic vocalizations reflect positive affect only, which may be not always the case (see, for example: 10.1016/j.neubiorev.2023.105260; 10.1007/s40732-025-00633-4; 10.1016/j.pnpbp.2020.110184). Therefore, the author must also present their data in the framework of the current evidence on the behavioral significance of ultrasonic vocalizations broadly speaking, and considering them not simply as indicators of positive affect. The same considerations apply to the paragraph “Conclusions”, where the last sentence must be toned down.

Discussion: “Furthermore, the absence or minimal change in the number of 50-kHz USVs, especially hedonic FM, which serves as an index of positive emotion and motivation [10, 11, 48], suggests that the rats’ emotional and motivational circuits were largely unaffected”. Again, this sentence is an overstatement that must be toned down. Please see my previous comments.

Reviewer #2: This study aims to investigate the role of the GABAergic system in the VPL in 50-kHz USV production and tactile reward-induced behavior. To address this question, adult rats are used, and behavioral assessments are conducted before and after muscimol injection. The manuscript has several major concerns that should be carefully addressed.

1. The manuscript lacks a clearly scientific rationale.

2. This study is largely descriptive. Incorporating mechanistic explanations/experiments would substantially strengthen the conclusions.

3. Substantial information is missing in the section of Methods. For example, the rationale for including only male rats should be clearly explained, given the potential sex differences in affective vocalization and reward-related behaviors.

4. More information is needed regarding the anatomical specificity of the muscimol injection. Please clarify how the drug diffusion was restricted to the VPL and whether adjacent thalamic nuclei might have been affected.

5. This manuscript lacks in-depth discussion. It is highly recommended to provide more comprehensive discussions, take-home messages, etc.

**********

6. PLOS authors have the option to publish the peer review history of their article (what does this mean?). If published, this will include your full peer review and any attached files.

If you choose “no”, your identity will remain anonymous but your review may still be made public.

**Do you want your identity to be public for this peer review?** For information about this choice, including consent withdrawal, please see our Privacy Policy.

Reviewer #1: No

Reviewer #2: No

**********

[NOTE: If reviewer comments were submitted as an attachment file, they will be attached to this email and accessible via the submission site. Please log into your account, locate the manuscript record, and check for the action link "View Attachments". If this link does not appear, there are no attachment files.]

To ensure your figures meet our technical requirements, please review our figure guidelines: https://journals.plos.org/plosone/s/figures

You may also use PLOS’s free figure tool, NAAS, to help you prepare publication quality figures: https://journals.plos.org/plosone/s/figures#loc-tools-for-figure-preparation.

NAAS will assess whether your figures meet our technical requirements by comparing each figure against our figure specifications.

# Author comment: Author response to Decision Letter 1

**DOI:** 10.1371/journal.pone.0351495.r002

25 Mar 2026

Response to Reviewer #1

Thank you for your valuable comments on our manuscript. We have revised the manuscript accordingly. Our responses to your comments are provided below.

Animals: please provide a clear description of the experimental groups. It is stated that 10 animals were used and that 4 animals were used per experiment. Please indicate how many experiments were performed, if rats were re-used and how. Moreover, it is reported that rats were individually housed. Since isolation can alter some dimension of ultrasonic vocalization in rats (see for example, 10.1016/j.bbr.2022.114157), it should be discussed whether and how individual housing may have influenced the results obtained.

Response:

Thank you for your comments. We corrected the number of rats used and added a discussion about the influence of individual housing (page 10, line 154; page 33–34, lines 569–584). A total of 12 rats were used in three experiments with four rats in each. Of all rats, two were used in a preliminary experiment, and excluded from data analysis and the remaining 10 were used. The original manuscript indicated four animals; we revised the text to indicate that there were 2–4 animals, excluding the two used in the preliminary experiment.

Surgery: please discuss whether the use of pentobarbital, which targets GABAergic receptors, may be expected to have any residual influences on the effects of muscimol evaluated at a later point. Moreover, please indicate which antibiotic therapy was used.

Response:

We revised the discussion accordingly (page 34, lines 585–592). The antibiotic therapy used in this study was specified in the text (page 11, lines 179–180).

Microinjection of GABA-A receptor agonists: “agonists” should be in singular form, since only muscimol was used. Moreover, the use of a single dose of muscimol should be acknowledged as a potential limitation of the present study.

Response:

Thank you for your comments. We revised the text accordingly and included the use of a single dose of muscimol as a limitation (page 11, line 164; page 12, line 183; page 35, lines 599–609).

Recording and analysis of ultrasonic vocalizations: “Specifically, trill, complex, and step up calls, which have been shown to be mediated by the accumbal dopamine system [10] are among the frequency-modulated (FM) calls that are indicators of positive emotions”. This sentence contains a series of overstatements. First, the quoted review article does not say that. Second trill, complex and step up calls are not the only to be mediated by accumbal dopamine system, since all the 50 kHz ultrasonic vocalizations depend on the accumbal dopamine system (see, for example: 10.1016/j.conb.2013.01.014; 10.1016/j.jneumeth.2018.06.018). Third, the use of those specific subtypes of ultrasonic vocalizations as indicators of positive emotions is a popular approach in the ultrasonic vocalization community, that however still lacks a final validation. Proof of this is the evidence from several studies by different groups that rats may emit “flat” 50 kHz ultrasonic vocalizations in situations that are believed to have positive emotional valence (see for example, 0.1016/j.pnpbp.2018.07.002; 10.1016/j.bbr.2018.02.041). Accordingly, the author must tone down these statements and discuss them in the framework of the existing literature on ultrasonic vocalizations. If the author wish to focus on the specific call subtypes reported, this may be fine, but the significance of those calls should be presented in a fair way, with reference to the existing literature, that must be acknowledged and discussed, and with reference to the earlier studies by the same author that investigated those specific call subtypes [i.e., references 10 and 12 quoted in the present manuscript).

Response:

Thank you for your insightful comments. We agree, so we corrected the text accordingly (page 13 line 206; page 21 line 345; page 27 lines 467–468).

Histological verification of injection sites: please clarify if the rats displaying a spread of dye to the VPM or RT were included in the study or excluded from the study.

Response:

Thank you for your comments. We revised the text accordingly (page 17, lines 287–288).

Bilateral muscimol injection into the VPL preserves affective 50-kHz USVs during and after rhythmic stroking: this paragraph and the associated figure must be reorganized. Again, the categorization of FM calls in “hedonic” and “other” is, at best, arbitrary. Again, no evidence exists to conclude that certain types of FM 50 kHz ultrasonic vocalizations communicate the presence of positive affect better than others, This is believed to occur for trill calls, but not all the available pieces of experimental evidence agree on this. Plus, on which basis were “split” calls dealt with as a separate category? Moreover, please define “harmonics”. Consider that according to some manufacturers of ultrasonic recording equipment (for example, Noldus) “harmonics” do not even exist as a real entity, rather being an artifact of the recording system used. Furthermore, this categorization in “hedonic” and “other” FM calls is also challenged by the very same results of this study that found an increase in flat calls. All this said, it is recommended that a new categorization of calls in flat and FM is used and presented, since the categorization proposed here may be criticized in many instances.

Response:

Thank you for your insightful and valuable comments. We revised the text accordingly (pages 3–5, lines 20–63; page 10, lines 149–152; page 13, lines 205–216; pages 21–22, lines 350–366; page 23, lines 380–382, 386–388; page 24, line 397; pages 30–33, lines 517–568). We agree on the lack of evidence to conclude that any specific subtype of 50 kHz USV conveys a positive emotional state better than other vocalizations. According to your comments, we present the results in two categories: FM and Flat with a focus on the specific call subtypes we reported and their underlying mechanisms. Based on the points you raised, we improved our classification method, unifying split and harmonics rather than separating them, and presented four categories. In addition, we referred to existing literature, recognized and discussed it, and referred to our previous studies investigating those specific call subtypes, ensuring that we do not deviate from the existing framework.

Discussion: “The results of this experiment may support the idea that thalamic dysfunction is linked to various psychiatric disorders, including schizophrenia, major depressive disorder, and autism spectrum disorder [53]”. Again, this sentence is an overstatement that is based on the hypothesis that 50 kHz ultrasonic vocalizations reflect positive affect only, which may be not always the case (see, for example: 10.1016/j.neubiorev.2023.105260; 10.1007/s40732-025-00633-4; 10.1016/j.pnpbp.2020.110184). Therefore, the author must also present their data in the framework of the current evidence on the behavioral significance of ultrasonic vocalizations broadly speaking, and considering them not simply as indicators of positive affect. The same considerations apply to the paragraph “Conclusions”, where the last sentence must be toned down.

Response:

Thank you for your valuable comments. We revised the text accordingly (page 27, lines 458–464; page 36, lines 626–628).

Discussion: “Furthermore, the absence or minimal change in the number of 50-kHz USVs, especially hedonic FM, which serves as an index of positive emotion and motivation [10, 11, 48], suggests that the rats’ emotional and motivational circuits were largely unaffected”. Again, this sentence is an overstatement that must be toned down. Please see my previous comments.

Response:

Thank you for your valuable comments. We revised the text accordingly (page 27, lines 467–468).

Response to Reviewer #2

Thank you for your valuable comments on our manuscript. We have revised the manuscript accordingly. Our responses to your comments are provided below.

1. The manuscript lacks a clearly scientific rationale.

Response:

We appreciate your point regarding the scientific rationale. We included background information about the use of ultrasonic vocalizations in the Introduction to strengthen the basis of this experiment (page 2, lines 2–3; pages 3–5, lines 20–63).

2. This study is largely descriptive. Incorporating mechanistic explanations/experiments would substantially strengthen the conclusions.

Response:

Thank you for your comments. We revised the text accordingly (pages 30–33, lines 517–568; pages 35–36, lines 594–619).

3. Substantial information is missing in the section of Methods. For example, the rationale for including only male rats should be clearly explained, given the potential sex differences in affective vocalization and reward-related behaviors.

Response:

Thank you for your comments. We revised the text accordingly (page 10, lines 149–152; page 35–36, lines 609–619).

4. More information is needed regarding the anatomical specificity of the muscimol injection. Please clarify how the drug diffusion was restricted to the VPL and whether adjacent thalamic nuclei might have been affected.

Response:

Thank you for your comments. We revised the text accordingly (page 7, lines 287–288; page 35, lines 594–599).

5. This manuscript lacks in-depth discussion. It is highly recommended to provide more comprehensive discussions, take-home messages, etc.

Response:

Thank you for your valuable comments. We revised the text accordingly (page 27, lines 458–464; page 36, lines 626–627). We agree that a deeper interpretation of our findings would strengthen the manuscript. Accordingly, we thoroughly revised the Discussion to provide a more in-depth analysis of the implications of our results. Specifically, we expanded on the mechanistic insights of our results and included a Conclusion that highlights the practical significance of our findings for the field. We believe these additional address the lack of depth and enhance the contribution of our work.

Attachment Submitted filename: Response to Reviewers.docx

# Peer review: Decision Letter 1

**DOI:** 10.1371/journal.pone.0351495.r003

**Authors:** Uwe Rudolph

15 Apr 2026

PONE-D-26-02461R1

Muscimol injection into the ventral posterolateral nucleus of the thalamus impairs tactile reward-seeking behavior but preserves affective vocalization in rats

PLOS One

Dear Dr. Shimoju,

Thank you for submitting your manuscript to PLOS ONE. After careful consideration, we feel that it has merit but does not fully meet PLOS ONE’s publication criteria as it currently stands. Therefore, we invite you to submit a revised version of the manuscript that addresses the points raised during the review process.

Please note that Reviewer 1 points out that it is absolutely necessary to describe the sample size adequately, and that a sample size of 4 is not sufficient for the experiments performed.

Upon request, Reviewer #2 provided more clarification to his :Review Comments to the Author":

For Question 1, a clear scientific rationale should be provided rather than listing background information.

*For Question 3, a justification for including only male subjects is required, rather than a descriptive statement.*

*For Question 4, the authors state: “Although slight diffusion was observed in some rats, all 10 rats were used in the data because the bilateral cannula tips were almost located in the target position.” It is unclear what is meant by “slight diffusion” and “almost”. What methods were used to assess or validate this? In addition, the histological results appear to show substantial tissue damage along the track.*

Please address these points clearly in your response.

Please submit your revised manuscript by May 30 2026 11:59PM. If you will need more time than this to complete your revisions, please reply to this message or contact the journal office at plosone@plos.org. When you're ready to submit your revision, log on to https://www.editorialmanager.com/pone/ and select the 'Submissions Needing Revision' folder to locate your manuscript file.

Please include the following items when submitting your revised manuscript:

- A letter that responds to each point raised by the academic editor and reviewer(s). You should upload this letter as a separate file labeled 'Response to Reviewers'.
- A marked-up copy of your manuscript that highlights changes made to the original version. You should upload this as a separate file labeled 'Revised Manuscript with Track Changes'.
- An unmarked version of your revised paper without tracked changes. You should upload this as a separate file labeled 'Manuscript'.

If you would like to make changes to your financial disclosure, please include your updated statement in your cover letter. Guidelines for resubmitting your figure files are available below the reviewer comments at the end of this letter.

If applicable, we recommend that you deposit your laboratory protocols in protocols.io to enhance the reproducibility of your results. Protocols.io assigns your protocol its own identifier (DOI) so that it can be cited independently in the future. For instructions see: https://journals.plos.org/plosone/s/submission-guidelines#loc-laboratory-protocols. Additionally, PLOS ONE offers an option for publishing peer-reviewed Lab Protocol articles, which describe protocols hosted on protocols.io. Read more information on sharing protocols at https://plos.org/protocols?utm_medium=editorial-email&utm_source=authorletters&utm_campaign=protocols.

As the corresponding author, your ORCID iD is verified in the submission system and will appear in the published article. PLOS supports the use of ORCID, and we encourage all coauthors to register for an ORCID iD and use it as well. Please encourage your coauthors to verify their ORCID iD within the submission system before final acceptance, as unverified ORCID iDs will not appear in the published article. *Only* the individual author can complete the verification step; PLOS staff *cannot* verify ORCID iDs on behalf of authors.

We look forward to receiving your revised manuscript.

Kind regards,

Uwe Rudolph

Academic Editor

PLOS One

Journal Requirements:

If the reviewer comments include a recommendation to cite specific previously published works, please review and evaluate these publications to determine whether they are relevant and should be cited. There is no requirement to cite these works unless the editor has indicated otherwise.

[Note: HTML markup is below. Please do not edit.]

Reviewers' comments:

Reviewer's Responses to Questions

**Comments to the Author**

1. If the authors have adequately addressed your comments raised in a previous round of review and you feel that this manuscript is now acceptable for publication, you may indicate that here to bypass the “Comments to the Author” section, enter your conflict of interest statement in the “Confidential to Editor” section, and submit your "Accept" recommendation.

Reviewer #1: (No Response)

Reviewer #2: (No Response)

**********

2. Is the manuscript technically sound, and do the data support the conclusions?

The manuscript must describe a technically sound piece of scientific research with data that supports the conclusions. Experiments must have been conducted rigorously, with appropriate controls, replication, and sample sizes. The conclusions must be drawn appropriately based on the data presented.

Reviewer #1: Partly

Reviewer #2: Partly

**********

3. Has the statistical analysis been performed appropriately and rigorously?

Reviewer #1: No

Reviewer #2: Yes

**********

4. Have the authors made all data underlying the findings in their manuscript fully available?

The PLOS Data policy requires authors to make all data underlying the findings described in their manuscript fully available without restriction, with rare exception (please refer to the Data Availability Statement in the manuscript PDF file). The data should be provided as part of the manuscript or its supporting information, or deposited to a public repository. For example, in addition to summary statistics, the data points behind means, medians and variance measures should be available. If there are restrictions on publicly sharing data—e.g. participant privacy or use of data from a third party—those must be specified.

Reviewer #1: Yes

Reviewer #2: Yes

**********

5. Is the manuscript presented in an intelligible fashion and written in standard English?

PLOS ONE does not copyedit accepted manuscripts, so the language in submitted articles must be clear, correct, and unambiguous. Any typographical or grammatical errors should be corrected at revision, so please note any specific errors here.

Reviewer #1: Yes

Reviewer #2: Yes

**********

6. Review Comments to the Author

Please use the space provided to explain your answers to the questions above. You may also include additional comments for the author, including concerns about dual publication, research ethics, or publication ethics. (Please upload your review as an attachment if it exceeds 20,000 characters)

Reviewer #1: The author has addressed most of my comments, but a critical issue remains which concerns the sample size for each experiment. Specifically: figure legends report that a total of 10 animals were used but do not report how many animals were used for each evaluation. The author replied that "A total of 12 rats were used in three experiments with four rats in each. Of all rats, two were used in a preliminary experiment, and excluded from data analysis and the remaining 10 were used. The original manuscript indicated four animals; we revised the text to indicate that there were 2–4 animals, excluding the two used in the preliminary experiment". However, it is still unclear how may animals were used in each evaluation. This must be clearly specified. Please note that a sample size of 4 would be too low, given the variability in ultrasonic vocalization among rats, and would dramatically reduce the robustness of data.

Reviewer #2: The author did not fully address my previous question. There is no clear improvement in the revised version.

**********

7. PLOS authors have the option to publish the peer review history of their article (what does this mean?). If published, this will include your full peer review and any attached files.

If you choose “no”, your identity will remain anonymous but your review may still be made public.

**Do you want your identity to be public for this peer review?** For information about this choice, including consent withdrawal, please see our Privacy Policy.

Reviewer #1: No

Reviewer #2: No

**********

[NOTE: If reviewer comments were submitted as an attachment file, they will be attached to this email and accessible via the submission site. Please log into your account, locate the manuscript record, and check for the action link "View Attachments". If this link does not appear, there are no attachment files.]

To ensure your figures meet our technical requirements, please review our figure guidelines: https://journals.plos.org/plosone/s/figures

You may also use PLOS’s free figure tool, NAAS, to help you prepare publication quality figures: https://journals.plos.org/plosone/s/figures#loc-tools-for-figure-preparation.

NAAS will assess whether your figures meet our technical requirements by comparing each figure against our figure specifications.

# Author comment: Author response to Decision Letter 2

**DOI:** 10.1371/journal.pone.0351495.r004

1 May 2026

Response to Editor

We appreciate the editor and reviewers for their valuable comments regarding our manuscript and have made revisions accordingly. The following are the responses to the editor’s comments. Revisions in the revised manuscript are in red font.

For specific Comments

1. Please note that Reviewer 1 points out that it is absolutely necessary to describe the sample size adequately, and that a sample size of 4 is not sufficient for the experiments performed.

Response:

We sincerely apologize for the misleading wording. We corrected the text to indicate that the number of samples used in the experiment was 10 (page 10–11, lines 156–165). Furthermore, as stated on page 15 (line 247), the study was conducted using a within-subject design; thus, all 10 rats received the vehicle and muscimol.

2. Upon request, Reviewer #2 provided more clarification to his: Review Comments to the Author":

For Question 1, a clear scientific rationale should be provided rather than listing background information.

For Question 3, a justification for including only male subjects is required, rather than a descriptive statement.

For Question 4, the authors state: “Although slight diffusion was observed in some rats, all 10 rats were used in the data because the bilateral cannula tips were almost located in the target position.” It is unclear what is meant by “slight diffusion” and “almost”. What methods were used to assess or validate this? In addition, the histological results appear to show substantial tissue damage along the track.

Please address these points clearly in your response.

Response:

Thank you for confirming that we could obtain more detailed information regarding reviewer #2's comments. We addressed each of your points in the response to reviewer #2.

(Questions 1: page 9, lines 136–139; Question 3: page 10, lines 152–156; page 36, lines 616–623; Question 4: page 35, lines 594–598, lines 602–603).

Journal Requirements:

If the reviewer comments include a recommendation to cite specific previously published works, please review and evaluate these publications to determine whether they are relevant and should be cited. There is no requirement to cite these works unless the editor has indicated otherwise.

Response:

Thank you for your explanation. We have carefully considered the comments provided by Reviewer #2 and evaluated their relevance to the scope and context of this study. In response, we have cited and incorporated literature that is both appropriate and pertinent to justifying the exclusive use of male rats. We have also ensured that the cited references represent a broad range of research findings in this field, while maintaining the specific focus of the present study.

Response to Reviewer #1

Thank you for your valuable comments. We have revised the manuscript accordingly, and our responses are provided below.

The author has addressed most of my comments, but a critical issue remains, which concerns the sample size for each experiment. Specifically: figure legends report that a total of 10 animals were used but do not report how many animals were used for each evaluation. The author replied that "A total of 12 rats were used in three experiments with four rats in each. Of all rats, two were used in a preliminary experiment, and excluded from data analysis and the remaining 10 were used. The original manuscript indicated four animals; we revised the text to indicate that there were 2–4 animals, excluding the two used in the preliminary experiment". However, it is still unclear how may animals were used in each evaluation. This must be clearly specified. Please note that a sample size of 4 would be too low, given the variability in ultrasonic vocalization among rats, and would dramatically reduce the robustness of data.

Response:

We would like to thank Reviewer #1 for indicating that our previous response did not fully clarify the sample size (n = 10). We apologize for the lack of clarity in the previous version and for not adequately addressing this point. To respond more appropriately, we have revised the text accordingly (page 10–11, lines 156–165).

Response to Reviewer #2

Thank you for your valuable comments. We have revised the manuscript accordingly and our responses are provided below.

Major comments

The author did not fully address my previous question. There is no clear improvement in the revised version.

Response:

We deeply appreciate your time in reviewing our manuscript. We apologize for not being able to adequately address your requests for feedback. Regarding the portions of Reviewer #2’s previous questions that we did not clearly answer, we provide our responses to the specific comments below that were received from the editor.

Specific comments

1. For Question 1, a clear scientific rationale should be provided rather than listing background information.

Response:

We thank Reviewer #2 for taking the time to review our manuscript. To add a clear scientific rationale, we added a section on novelty, knowledge gaps, and the significance of the study to the final paragraph of the Introduction (page 9, lines 136–139). We hope that our understanding and this revision satisfy Reviewer #2’s points. If the revision is not consistent with Reviewer #2's views, we would appreciate additional feedback. Reviewer #2 indicated that we need “scientific rationale” rather than just a list of references. Does this mean reducing the background explanation or focusing directly on the research topic? We would like to confirm whether the revisions meet your expectations. Notably, when using rat ultrasonic vocalizations as a behavioral indicator, as in this study, it is necessary to explain to the reader that rat ultrasonic vocalizations are an indicator of positive emotions, that there are problems with the classification method of ultrasonic vocalizations (within the scope of Reviewer #1’s points), that rhythmic stroking is a stimulation method unique to our laboratory, and that we are studying a niche topic, which is the effect of somatosensory stimulation on emotion. This inevitably makes the background explanation lengthy. We would appreciate it if more specific feedback could be provided on the lack of scientific rationale as we are prepared to make revisions accordingly.

2. For Question 3, a justification for including only male subjects is required, rather than a descriptive statement.

Response:

Thank you for your valuable comments. We have revised the text to justify the use of only male rats (page 10, lines 152–156; page 36, lines 616–623).

3. For Question 4, the authors state: “Although slight diffusion was observed in some rats, all 10 rats were used in the data because the bilateral cannula tips were almost located in the target position.” It is unclear what is meant by “slight diffusion” and “almost”. What methods were used to assess or validate this? In addition, the histological results appear to show substantial tissue damage along the track.

Response:

Thank you for your valuable comments. In this study, we examined the localization of Evans Blue (EB) administration to the VPL by observing its diffusion under a light microscope. We acknowledge the limitation that cellular-level effects were not assessed using immunohistochemical techniques (page 35, lines 594–598). In addition, we have removed sections that provided vague descriptions of EB diffusion, as this was not the primary focus of the study (page 18, lines 288–292).

In the microinjection experiment, the degree of tissue damage caused by guide cannula placements is usually present to the extent observed in this study. However, we felt that it was necessary to show that there was no significant effect on the USV before and after tissue damage, and therefore, we added a supplementary fig. S2 (page 20, lines 331–335).

Attachment Submitted filename: Response to Reviewer.docx

# Peer review: Decision Letter 2

**DOI:** 10.1371/journal.pone.0351495.r005

**Authors:** Uwe Rudolph

18 May 2026

PONE-D-26-02461R2

Muscimol injection into the ventral posterolateral nucleus of the thalamus impairs tactile reward-seeking behavior but preserves affective vocalization in rats

PLOS One

Dear Dr. Shimoju,

Thank you for submitting your manuscript to PLOS ONE. After careful consideration, we feel that it has merit but does not fully meet PLOS ONE’s publication criteria as it currently stands. Therefore, we invite you to submit a revised version of the manuscript that addresses the points raised during the review process.

==============================

**ACADEMIC EDITOR:**

I agree with reviewer #2 that since only male animals were used, the title should be changed to contain the word 'male' ('....but preserves affective vocalizations in rats').  If the author has a specific justification for only using male mice, this could be added.

==============================

Please submit your revised manuscript by Jul 02 2026 11:59PM. If you will need more time than this to complete your revisions, please reply to this message or contact the journal office at plosone@plos.org. When you're ready to submit your revision, log on to https://www.editorialmanager.com/pone/ and select the 'Submissions Needing Revision' folder to locate your manuscript file.

Please include the following items when submitting your revised manuscript:

- A letter that responds to each point raised by the academic editor and reviewer(s). You should upload this letter as a separate file labeled 'Response to Reviewers'.
- A marked-up copy of your manuscript that highlights changes made to the original version. You should upload this as a separate file labeled 'Revised Manuscript with Track Changes'.
- An unmarked version of your revised paper without tracked changes. You should upload this as a separate file labeled 'Manuscript'.

If you would like to make changes to your financial disclosure, please include your updated statement in your cover letter. Guidelines for resubmitting your figure files are available below the reviewer comments at the end of this letter.

If applicable, we recommend that you deposit your laboratory protocols in protocols.io to enhance the reproducibility of your results. Protocols.io assigns your protocol its own identifier (DOI) so that it can be cited independently in the future. For instructions see: https://journals.plos.org/plosone/s/submission-guidelines#loc-laboratory-protocols. Additionally, PLOS ONE offers an option for publishing peer-reviewed Lab Protocol articles, which describe protocols hosted on protocols.io. Read more information on sharing protocols at https://plos.org/protocols?utm_medium=editorial-email&utm_source=authorletters&utm_campaign=protocols.

As the corresponding author, your ORCID iD is verified in the submission system and will appear in the published article. PLOS supports the use of ORCID, and we encourage all coauthors to register for an ORCID iD and use it as well. Please encourage your coauthors to verify their ORCID iD within the submission system before final acceptance, as unverified ORCID iDs will not appear in the published article. *Only* the individual author can complete the verification step; PLOS staff *cannot* verify ORCID iDs on behalf of authors.

We look forward to receiving your revised manuscript.

Kind regards,

Uwe Rudolph

Academic Editor

PLOS One

**Journal Requirements:**

1. If the reviewer comments include a recommendation to cite specific previously published works, please review and evaluate these publications to determine whether they are relevant and should be cited. There is no requirement to cite these works unless the editor has indicated otherwise.

2. Please review your reference list to ensure that it is complete and correct. If you have cited papers that have been retracted, please include the rationale for doing so in the manuscript text, or remove these references and replace them with relevant current references. Any changes to the reference list should be mentioned in the rebuttal letter that accompanies your revised manuscript. If you need to cite a retracted article, indicate the article’s retracted status in the References list and also include a citation and full reference for the retraction notice.

[Note: HTML markup is below. Please do not edit.]

Reviewers' comments:

Reviewer's Responses to Questions

**Comments to the Author**

1. If the authors have adequately addressed your comments raised in a previous round of review and you feel that this manuscript is now acceptable for publication, you may indicate that here to bypass the “Comments to the Author” section, enter your conflict of interest statement in the “Confidential to Editor” section, and submit your "Accept" recommendation.

Reviewer #1: All comments have been addressed

Reviewer #2: (No Response)

**********

2. Is the manuscript technically sound, and do the data support the conclusions?

The manuscript must describe a technically sound piece of scientific research with data that supports the conclusions. Experiments must have been conducted rigorously, with appropriate controls, replication, and sample sizes. The conclusions must be drawn appropriately based on the data presented.

Reviewer #1: Yes

Reviewer #2: (No Response)

**********

3. Has the statistical analysis been performed appropriately and rigorously?

Reviewer #1: Yes

Reviewer #2: (No Response)

**********

4. Have the authors made all data underlying the findings in their manuscript fully available?

The PLOS Data policy requires authors to make all data underlying the findings described in their manuscript fully available without restriction, with rare exception (please refer to the Data Availability Statement in the manuscript PDF file). The data should be provided as part of the manuscript or its supporting information, or deposited to a public repository. For example, in addition to summary statistics, the data points behind means, medians and variance measures should be available. If there are restrictions on publicly sharing data—e.g. participant privacy or use of data from a third party—those must be specified.

Reviewer #1: Yes

Reviewer #2: (No Response)

**********

5. Is the manuscript presented in an intelligible fashion and written in standard English?

PLOS ONE does not copyedit accepted manuscripts, so the language in submitted articles must be clear, correct, and unambiguous. Any typographical or grammatical errors should be corrected at revision, so please note any specific errors here.

Reviewer #1: Yes

Reviewer #2: (No Response)

**********

6. Review Comments to the Author

Please use the space provided to explain your answers to the questions above. You may also include additional comments for the author, including concerns about dual publication, research ethics, or publication ethics. (Please upload your review as an attachment if it exceeds 20,000 characters)

Reviewer #1: The author has addressed all my concerns. The description of experimental groups is now clear. I have no further comments.

Reviewer #2: For Q3, there is still no explanation for why only male subjects were used. I would recommend adding “male” to the title to avoid misleading the audience.

**********

7. PLOS authors have the option to publish the peer review history of their article (what does this mean?). If published, this will include your full peer review and any attached files.

If you choose “no”, your identity will remain anonymous but your review may still be made public.

**Do you want your identity to be public for this peer review?** For information about this choice, including consent withdrawal, please see our Privacy Policy.

Reviewer #1: No

Reviewer #2: No

**********

[NOTE: If reviewer comments were submitted as an attachment file, they will be attached to this email and accessible via the submission site. Please log into your account, locate the manuscript record, and check for the action link "View Attachments". If this link does not appear, there are no attachment files.]

To ensure your figures meet our technical requirements, please review our figure guidelines: https://journals.plos.org/plosone/s/figures

You may also use PLOS’s free figure tool, NAAS, to help you prepare publication quality figures: https://journals.plos.org/plosone/s/figures#loc-tools-for-figure-preparation.

NAAS will assess whether your figures meet our technical requirements by comparing each figure against our figure specifications.

# Author comment: Author response to Decision Letter 3

**DOI:** 10.1371/journal.pone.0351495.r006

27 May 2026

Response to Editor:

We appreciate the editor and reviewers for their valuable comments regarding our manuscript. We have revised the manuscript accordingly. Following are the responses to the editor’s comments. Please note that the revisions are indicated in red font.

ACADEMIC EDITOR:

I agree with reviewer #2 that since only male animals were used, the title should be changed to contain the word 'male' ('....but preserves affective vocalizations in rats'). If the author has a specific justification for only using male mice, this could be added.

Response:

Thank you for your valuable comments. We agree that it should be clearly stated to the readers that only male rats were used in study. Accordingly, we have updated the title to “Muscimol injection into the ventral posterolateral nucleus of the thalamus impairs tactile reward-seeking behavior but preserves affective vocalization in male rats.” The reason for using only male animals is that the effects of changes in psychosomatic functions associated with fluctuations in the estrous cycle of female rats on emotions during tactile stimulation are still unknown, and thus, experiments using female rats would be beyond the scope of this research topic. This point has already been described in the methods and research limitations sections (page 10, lines 152–156; page 36, lines 612–623).

Journal Requirements:

1. If the reviewer comments include a recommendation to cite specific previously published works, please review and evaluate these publications to determine whether they are relevant and should be cited. There is no requirement to cite these works unless the editor has indicated otherwise.

Response:

Thank you for your valuable comment. We have carefully considered the comments provided by Reviewer #2 and evaluated their relevance to the scope and context of this study. The reviewers’ comments did not include any recommendations to cite specific previously published literature, and therefore, I did not cite any literature separately.

2. Please review your reference list to ensure that it is complete and correct. If you have cited papers that have been retracted, please include the rationale for doing so in the manuscript text, or remove these references and replace them with relevant current references. Any changes to the reference list should be mentioned in the rebuttal letter that accompanies your revised manuscript. If you need to cite a retracted article, indicate the article’s retracted status in the References list and also include a citation and full reference for the retraction notice.

Response:

We appreciate your insightful comments. We have confirmed that no retracted articles are included and that all references are complete and accurate.

Response to Reviewer #1

Thank you for your valuable comments.

Major comments

The author has addressed all my concerns. The description of experimental groups is now clear. I have no further comments.

Response:

Thank you for taking the time to review our manuscript, providing constructive feedback, and conducting a thorough review of the manuscript. We are also grateful for your assessment that we were able to adequately address the points you raised in the revisions.

Response to Reviewer #2

Thank you for your valuable comments. We have revised the manuscript accordingly and our responses are provided below.

Specific comments

there is still no explanation for why only male subjects were used. I would recommend adding “male” to the title to avoid misleading the audience.

Response:

We are grateful to you for this constructive feedback and for the specific guidance. We agree that it should be clearly communicated to readers that only male rats were used. As such, we have updated the title to “Muscimol injection into the ventral posterolateral nucleus of the thalamus impairs tactile reward-seeking behavior but preserves affective vocalization in male rats.” We sincerely apologize that we were unable to provide a response that met your expectations. Although we have stated our reasoning and research limitations (page 10, lines 152–156; page 36, lines 612–623), we would be extremely grateful for any further guidance you may have. Thank you very much for taking the time to review our paper.

Attachment Submitted filename: Response_to_Reviewers_auresp_3.docx

# Editor report: Decision Letter 3

**DOI:** 10.1371/journal.pone.0351495.r007

**Authors:** Uwe Rudolph

29 May 2026

Muscimol injection into the ventral posterolateral nucleus of the thalamus impairs tactile reward-seeking behavior but preserves affective vocalization in male rats

PONE-D-26-02461R3

Dear Dr. Shimoju,

We’re pleased to inform you that your manuscript has been judged scientifically suitable for publication and will be formally accepted for publication once it meets all outstanding technical requirements.

Within one week, you’ll receive an e-mail detailing the required amendments. When these have been addressed, you’ll receive a formal acceptance letter and your manuscript will be scheduled for publication.

An invoice will be generated when your article is formally accepted. Please note, if your institution has a publishing partnership with PLOS and your article meets the relevant criteria, all or part of your publication costs will be covered. Please make sure your user information is up-to-date by logging into Editorial Manager at Editorial Manager® and clicking the ‘Update My Information' link at the top of the page. For questions related to billing, please contact billing support.

If your institution or institutions have a press office, please notify them about your upcoming paper to help maximize its impact. If they’ll be preparing press materials, please inform our press team as soon as possible -- no later than 48 hours after receiving the formal acceptance. Your manuscript will remain under strict press embargo until 2 pm Eastern Time on the date of publication. For more information, please contact onepress@plos.org.

Kind regards,

Uwe Rudolph

Academic Editor

PLOS One

Additional Editor Comments (optional):

Reviewers' comments:

# Editor report: Acceptance letter

**DOI:** 10.1371/journal.pone.0351495.r008

**Authors:** Uwe Rudolph

PONE-D-26-02461R3

PLOS One

Dear Dr. Shimoju,

I'm pleased to inform you that your manuscript has been deemed suitable for publication in PLOS One. Congratulations! Your manuscript is now being handed over to our production team.

At this stage, our production department will prepare your paper for publication. This includes ensuring the following:

* All references, tables, and figures are properly cited

* All relevant supporting information is included in the manuscript submission,

* There are no issues that prevent the paper from being properly typeset

You will receive further instructions from the production team, including instructions on how to review your proof when it is ready. Please keep in mind that we are working through a large volume of accepted articles, so please give us a few days to review your paper and let you know the next and final steps.

Lastly, if your institution or institutions have a press office, please let them know about your upcoming paper now to help maximize its impact. If they'll be preparing press materials, please inform our press team within the next 48 hours. Your manuscript will remain under strict press embargo until 2 pm Eastern Time on the date of publication. For more information, please contact onepress@plos.org.

You will receive an invoice from PLOS for your publication fee after your manuscript has reached the completed accept phase. If you receive an email requesting payment before acceptance or for any other service, this may be a phishing scheme. Learn how to identify phishing emails and protect your accounts at https://explore.plos.org/phishing.

If we can help with anything else, please email us at customercare@plos.org.

Thank you for submitting your work to PLOS ONE and supporting open access.

Kind regards,

PLOS ONE Editorial Office Staff

on behalf of

Dr. Uwe Rudolph

Academic Editor

PLOS One
